# A tripartite cytolytic toxin formed by *Vibrio cholerae* proteins with flagellum-facilitated secretion

**DOI:** 10.1073/pnas.2111418118

**Published:** 2021-11-19

**Authors:** Aftab Nadeem, Raghavendra Nagampalli, Eric Toh, Athar Alam, Si Lhyam Myint, Thomas V. Heidler, Mitesh Dongre, Nikola Zlatkov, Hudson Pace, Fouzia Bano, Anders Sjöstedt, Marta Bally, Bernt Eric Uhlin, Sun Nyunt Wai, Karina Persson

**Affiliations:** ^a^Department of Molecular Biology, Umeå University, Umeå SE-90187, Sweden;; ^b^Laboratory for Molecular Infection Medicine Sweden, Umeå University, Umeå SE-90187, Sweden;; ^c^Umeå Centre for Microbial Research, Umeå University, Umeå SE-90187, Sweden;; ^d^Department of Chemistry, Umeå University, Umeå SE-90187, Sweden;; ^e^Department of Clinical Microbiology, Umeå University, Umeå SE-90185, Sweden;; ^f^Wallenberg Centre for Molecular Medicine, Umeå University, Umeå SE-90185, Sweden

**Keywords:** *Vibrio cholerae*, tripartite toxin, crystal structure

## Abstract

The protein MakA was discovered as a motility-associated secreted toxin from *Vibrio cholerae*. Here, we show that MakA is part of a gene cluster encoding four additional proteins: MakB, MakC, MakD, and MakE. MakA, MakB, and MakE were readily detected in culture supernatants of wild-type *V. cholerae*, whereas secretion was very much reduced from a flagellum-deficient mutant. Crystal structures of MakA, MakB, and MakE revealed a structural relationship to a superfamily of bacterial pore-forming toxins. Expression of MakA/B/E in *Escherichia coli* resulted in toxicity toward *Caenorhabditis elegans* used as a predatory model organism. None of these Mak proteins alone or in pairwise combinations were cytolytic, but an equimolar mixture of MakA, MakB, and MakE acted as a tripartite cytolytic toxin in vitro, causing lysis of erythrocytes and cytotoxicity on cultured human colon carcinoma cells. Formation of oligomeric complexes on liposomes was observed by electron microscopy. Oligomer interaction with membranes was initiated by MakA membrane binding followed by MakB and MakE joining the assembly of a pore structure. A predicted membrane insertion domain of MakA was shown by site-directed mutagenesis to be essential for toxicity toward *C. elegans*. Bioinformatic analyses revealed that the *makCDBAE* gene cluster is present as a genomic island in the vast majority of sequenced genomes of *V. cholerae* and the fish pathogen *Vibrio anguillarum*. We suggest that the hitherto-unrecognized cytolytic MakA/B/E toxin can contribute to Vibrionaceae fitness and virulence potential in different host environments and organisms.

## Significance

*Vibrio cholerae*, responsible for outbreaks of cholera disease, is a highly motile organism by virtue of a single flagellum. We describe how the flagellum facilitates the secretion of three *V. cholerae* proteins encoded by a hitherto-unrecognized genomic island. The proteins MakA/B/E can form a tripartite toxin that lyses erythrocytes and is cytotoxic to cultured human cells. A structural basis for the cytolytic activity of the Mak proteins was obtained by X-ray crystallography. Flagellum-facilitated secretion ensuring spatially coordinated delivery of Mak proteins revealed a role for the *V. cholerae* flagellum considered of particular significance for the bacterial environmental persistence. Our findings will pave the way for the development of diagnostics and therapeutic strategies against pathogenic Vibrionaceae.

*Vibrio cholerae* is known as the cause of cholera, a disease that can lead to fatal dehydration (1). The disease is caused by a few serogroups, and the main factor behind the symptoms is the cholera toxin (CT) encoded by genes located on a prophage mobile genetic element (CTX-φ) that induce severe disruption of intestinal cell function, leading to watery, secretory diarrhea (2). Most serogroups do not cause cholera, as they do not possess the genes for CT, but they cause other diseases [e.g., skin, wound, and gastrointestinal infections as well as bacteremia (3)]. The natural reservoirs of *V. cholerae* are aquatic sources such as rivers, brackish waters, and estuaries and are often associated with copepods, aquatic plants, and shellfish (4). The factors and mechanisms allowing *V. cholerae* and other Vibrionaceae to survive and thrive in harsh natural environments are of major interest to researchers (5).

*V. cholerae* is motile by virtue of a single polar flagellum. The flagellum export machinery and the virulence-associated type-III secretion system (fT3SS and vT3SS, respectively) are suggested to share a common ancestor (6), explaining their similar structure and molecular organization. The vT3SS allows the delivery of effector proteins through a hollow channel directly to the eukaryotic host cell (7), and flagellar proteins are delivered via the fT3SS channel during flagellum assembly. In the bacterial cytoplasm, effectors secreted by the vT3SS are stabilized by chaperones to prevent aggregation. These chaperones are often encoded by genes adjacent to those encoding the effectors (8). Flagellar proteins are similarly protected by chaperones before they are transported to the growing distal end of the flagellum (9).

We use *Caenorhabditis elegans* as a predatory organism model for identifying and assessing *V. cholerae* factors, other than CT, that may contribute to bacterial survival and persistence (10). With this model, we discovered a cytotoxin, MakA (motility-associated killing factor A), which we demonstrated to be an essential factor for the cytotoxic activity of *V. cholerae* in both *C. elegans* and *Danio rerio* (zebrafish) (11). We also demonstrated that secretion of MakA occurs via the flagellum in a manner that is undocumented in *V. cholerae*.

 Our crystal structure of MakA revealed similarities to ClyA (11), the pore-forming toxin first identified in nonpathogenic *Escherichia coli* (12, 13) and, subsequently, also in *Salmonella enterica* (14). ClyA from *E. coli* is expressed from a monocistronic operon and oligomerizes into a dodecameric pore upon release via membrane vesicles (13, 15, 16). MakA is also structurally related to two proteins from *Bacillus cereus*, the hemolysin BL binding component B (HBL-B) and the NheA component of the Nhe nonhemolytic enterotoxin. Both of these are considered components of tripartite toxins (17). Recently, a tripartite toxin, AhlABC, was identified and structurally characterized as a pore-forming toxin in *Aeromonas hydrophila,* and the structure of soluble AhlB shares the general structure described for MakA (18). A similar toxin complex of three proteins, SmhABC from *Serratia marcescens*, was also reported (19). However, if and how the Ahl and Smh proteins are released during normal growth, or if there is a dedicated secretion system, remain unclear.

Here, we identify the proteins from the five *V. cholerae* genes, *vca0880* through *vca0884*, that are coexpressed from the operon *makDCBAE* and analyze the crystal structures of MakA, MakB, and MakE. Our in vitro studies revealed that an equimolar combination of the MakA/B/E proteins acted as a tripartite cytotoxin causing lysis of red blood cells and cytotoxicity to epithelial cells. Examination of a large number of bacterial genomes revealed that the *mak* operon is present in many *V. cholerae* and other Vibrionaceae strains. These include *Vibrio (Listonella) anguillarum*, an inhabitant of estuarine and marine coastal ecosystems worldwide and the etiological agent of vibriosis in warm- and cold-water fish (20). The identification and structural characterization of the Mak proteins in *V. cholerae* presented here reveals a hitherto-unrecognized potential of many pathogenic Vibrionaceae strains to produce the tripartite Mak cytolytic toxin.

## Results

### Expression and Secretion of MakA, MakB, and MakE from *V. cholerae*.

**Fig. 1.**
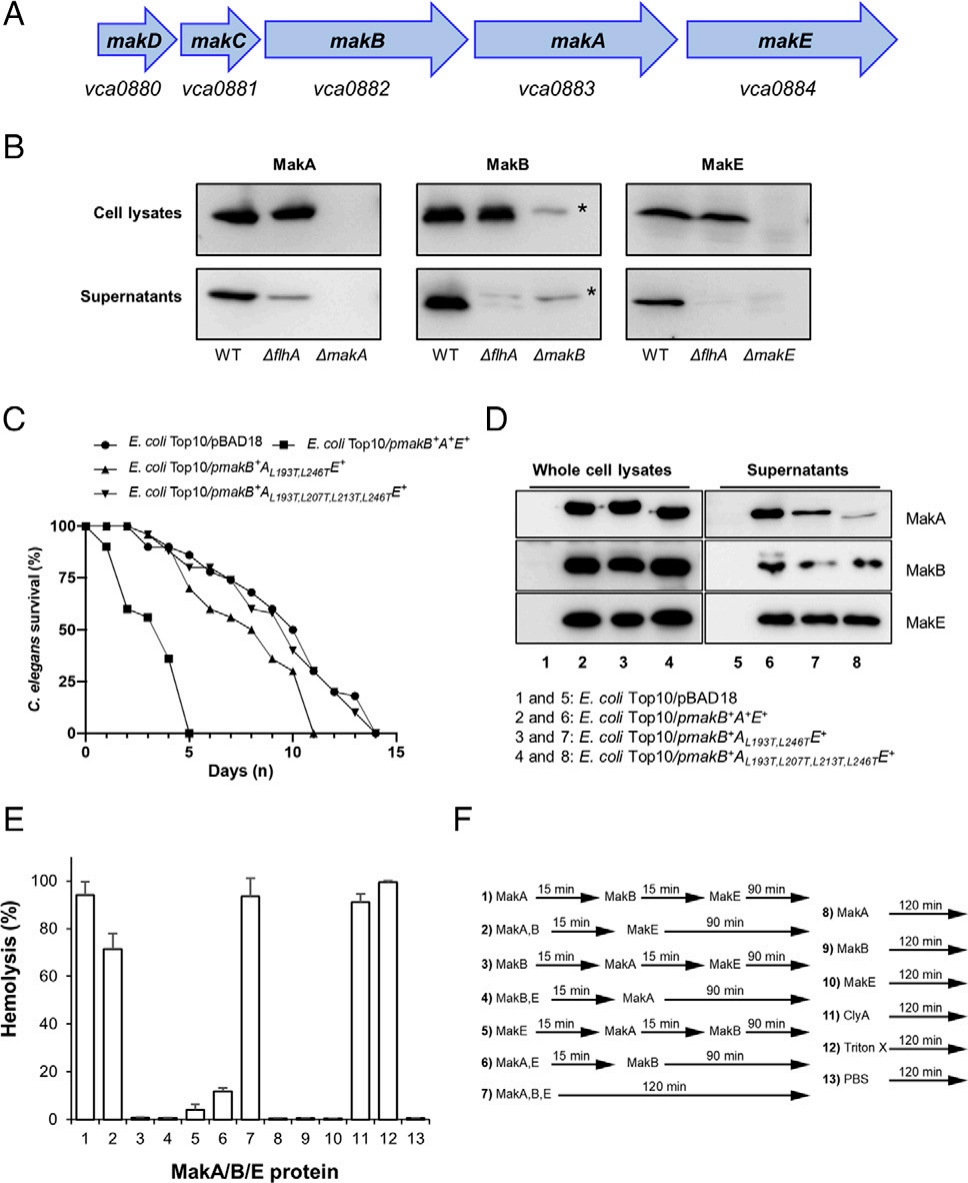
Secretion of Mak proteins forming a tripartite cytolytic toxin. (*A*) Gene organization of the *mak* operon in *V. cholerae* O1 El Tor strain A1552. The gene loci *vca0880*, *vca0881*, *vca0882*, *vca0883*, and *vca0884* have been denoted *makD*, *makC*, *makB*, *makA*, and *makE*, respectively. (*B*) Western immunoblotting was used to detect MakA, MakB, and MakE in whole-cell lysates and the supernatant. The study was done on wild-type *V. cholerae* A1552, Δ*flhA*, Δ*makA*, *ΔmakB*, and *ΔmakE* mutants. The proteins were detected with antisera raised against MakA, MakB, and MakE, respectively. The asterisk indicates an unidentified protein also detected by the MakB antiserum. (*C*) *C. elegans* survival upon feeding on *E. coli* Top10 harboring *makABE* genes and mutants thereof. Data represent average survival percentage of three separate experiments. (*D*) Western blot analysis of MakA, MakB, or MakE secreted to the culture supernatant or in whole-cell lysates of *E. coli* Top10 expressing wild-type MakA or MakA mutants together with MakB and MakE. (*E* and *F*) Lysis of erythrocytes by Mak proteins in solution. A total of 250 nM MakA, MakB, and MakE was added to human erythrocyte (2% whole blood) suspensions and incubated at 37 °C for 120 min, either individually, sequentially, or in combination. ClyA (250 nM) was used as a positive control and phosphate-buffered saline (PBS) as the negative control. Hemolysis was examined using spectrophotometry. Data are from three independent experiments.

We previously described a gene cluster in *V. cholerae* O1 strain A1552 comprising the genes *makD*, *makC*, *makB*, and *makA* (11). Later, an additional gene, *makE*, was identified downstream of *makA*. The five genes in the gene cluster are transcribed in the direction *makD → makC* → *makB → makA → makE* (Fig. 1*A*). By cloning and mutagenesis of each gene, we analyzed the encoded proteins. Previous tests with *V. cholerae* mutants defective in *makA* or *makB* demonstrated a clear attenuation of toxicity in *C. elegans*, whereas the effect of Δ*makD* or Δ*makC* was minimal (11).

MakA, composed of 369 amino acids, is mainly secreted via the *V. cholerae* flagellum (11). To test if MakB and MakE (354 and 353 amino acids, respectively) also are secreted, we analyzed samples from cell lysates and supernatants using anti-MakA, anti-MakB, and anti-MakE antisera (Fig. 1*B*). Similar to the earlier findings with MakA, both MakB and MakE were detected in the supernatant. When MakA, MakB, and MakE were expressed by a flagellum-deficient (Δ*flhA*) mutant of *V. cholerae*, the secretion of all three proteins was drastically reduced (Fig. 1*B*). We therefore conclude that secretion of these Mak proteins is facilitated by the *V. cholerae* flagellum. It should be noted that MakA secretion occurred to a level of about 10% even from the Δ*flhA* mutant derivative (11).

We monitored the expression and secretion of Mak proteins in *V. cholerae* derivatives mutated in each of the *mak* genes (*SI Appendix*, Fig. S1). There was a strong reduction in the cellular MakA levels in the *ΔmakB* mutant, and, consequently, very little MakA was secreted. Also, the Δ*makC*, Δ*makD*, and Δ*makE* mutants showed a somewhat lower cellular level of MakA, and, accordingly, the levels were lower in the supernatants (*SI Appendix*, Fig. S1*A*, lanes 3 and 9 through 12). The low MakA amount in the Δ*makB* mutant remained also when the strain was complemented in trans with a plasmid expressing MakB (*SI Appendix*, Fig. S1*E*). Similarly, MakB expression and secretion levels were much reduced in the Δ*makC* mutant (*SI Appendix*, Fig. S1 *B* and *D*, lanes 4 and 10). In addition, the amount of MakB in the Δ*makC* mutant remained low in the strain complemented with a plasmid expressing MakC (*SI Appendix*, Fig. S1*F*). The results indicated that deletions of *makB* or *makC* caused strong polarity effects, in particular on their immediate downstream neighboring genes. We did not detect any secretion of MakC (*SI Appendix*, Fig. S1*D*), and we consider that both MakC and MakD have accessory roles and remain in the bacterial cytoplasm or periplasm.

The *Vibrio* flagellum is covered by a sheath that surrounds the filament (21, 22). Secretion of the antisigma factor FlgM through the filament was demonstrated, which led to the suggestion that there may be a sheath opening at the flagellar tip. Studies suggest that the H-ring is essential for outer membrane penetration and assembly of the flagellum (23). Vibrios lacking FlgT (an H-ring component) synthesize some flagella that failed to penetrate the outer membrane, forming periplasmic flagella, and it was considered that the H-ring might play a role in sheath formation. In the absence of any known mutant lacking the sheath per se, we tested if secretion of the MakA/B/E proteins was affected by a Δ*flgT* mutation. The Δ*flgT* mutation did not cause any difference in secretion of the MakA/B/E proteins compared to the wild-type strain (*SI Appendix*, Fig. S2). Evidently, the proposed role of the H-ring to facilitate outer membrane penetration of the sheathed flagellum was not essential for Mak secretion.

The fact that MakB and MakE were also secreted in a flagella-facilitated manner prompted us to test if they also could be secreted from *E. coli* as was shown earlier for MakA (11). Using a plasmid with an inducible promoter comprising the *makB^+^*, *makA^+^*, and *makE^+^* genes, we tested if it mediated a toxic effect on *C. elegans,* similar to what we previously observed for MakA. As shown in Fig. 1*C* and *D* (lane 2), when MakA/B/E were expressed in *E. coli*, all three proteins were readily secreted into the supernatant and caused killing of *C. elegans.* We therefore conclude that the other two proteins, MakC and MakD from the *mak* operon, are not essential for the secretion of MakA/B/E and their toxin activity.

### The MakA/B/E Tripartite Has Hemolytic and Cytotoxic Activity.

We tested if recombinant MakA, MakB, and MakE as individual proteins or in bipartite/tripartite combinations would cause erythrocyte lysis by applying them on blood agar plates (*SI Appendix*, Fig. S3*A*). As positive control, we used the cytolytic protein ClyA (12, 13). We observed a clear hemolytic effect of the equimolar combination of the tripartite MakA/B/E but none for the individual proteins or bipartite combinations.

The cytolytic activity was further assessed by quantitative hemolysis assays with erythrocytes in solution (Fig. 1 *E* and *F*). The proteins (each at 250 nM) were introduced in different sequential orders over 120 min (Fig. 1 *E* and *F*). Maximum hemolytic activity was observed with the order MakA → MakB → MakE with intervals of 15 min (Fig. 1*E*, column 1) and when all three components were premixed prior to their addition to the erythrocytes (Fig. 1*E*, column 7). Notably, the hemolytic activity of the equimolar MakA/B/E tripartite was similar to that observed with 250 nM ClyA (Fig. 1*E*, column 11). Hemolysis, to about a 10 to 15% lower level, was also observed when MakA and MakB were first introduced as a premix and MakE was added last (Fig. 1*E*, column 2). Interestingly, when MakB was added first, followed by MakA and, finally, MakE, no hemolysis was detected within the 120-min assay (Fig. 1*E*, column 3). Similarly, when MakB and MakE were first introduced as a premix and MakA was added 15 min later, there was no detectable hemolysis (Fig. 1*E*, column 4). A low level of hemolysis (5 to 10%) was detected when the proteins were added separately in the order MakE → MakA → MakB or in the order MakAE → MakB (Fig. 1, columns 5 and 6, respectively). The individual Mak proteins did not cause any detectable hemolysis (Fig. 1, columns 8 through 10). We conclude that all three proteins were required for cytolytic activity and that there was a clear dependence on the order of addition. Only when MakA was added first, alone or in a premix with MakB, did the tripartite yield high-level hemolysis.

**Fig. 2.**
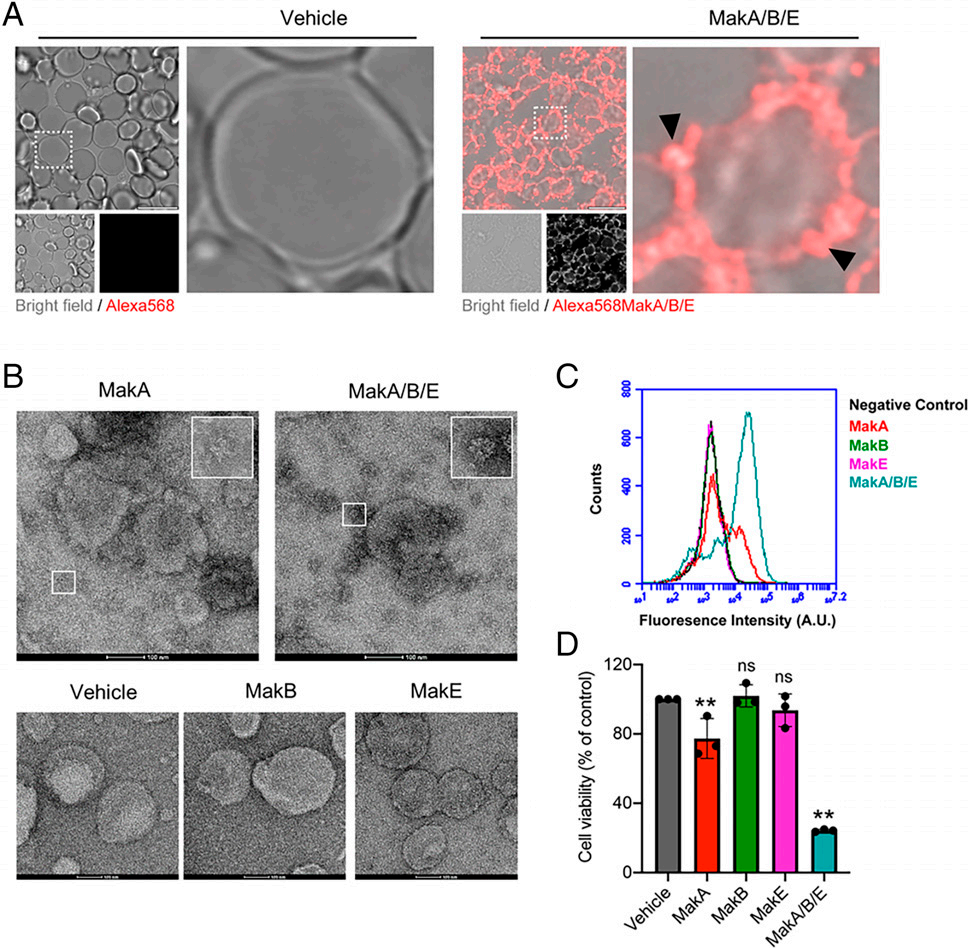
Activity of the tripartite MakA, MakB, and MakE cytotoxin on human cells. (*A*) Human red blood cells (1% whole blood) in PBS were treated with an Alexa568-labeled MakA, MakB, and MakE tripartite combination (125 nM of each). Binding and membrane accumulation of the Alexa568-labeled proteins were assessed by confocal laser scanning microscopy. Arrowheads (black) indicate membrane accumulation of Alexa568-MakA/B/E tripartite complex. (Scale bars, 10 µm.) (*B*) Liposomes from *E. coli* total LE were treated with vehicle (Tris 20 mM), MakA, MakB, MakE, or the MakA/B/E combination for 90 min and stained with 1.5% uranyl acetate solution. Micrographs were captured with TEM. Inset indicates star-shaped oligomers of MakA and larger complexes of the MakA/B/E tripartite complex. (Scale bars, 100 nm.) (*C*) Caco-2 cells were treated with Alexa568-labeled MakA, MakB, and MakE (250 nM of each) either individually or in the tripartite combination at equimolar concentrations. The cell-associated Alexa568-labeled protein was assessed after 24 h by flow cytometry analysis. (*D*) Caco-2 cells were treated with MakA, MakB, and MakE proteins (250 nM of each), either individually or in the tripartite combination, for 24 h, and toxicity was assessed by the MTS cell viability assay. Data points represent four biologically independent experiments; bar graphs show mean ± SD. Significance was determined from biological replicates using one-way ANOVA with Dunnett’s multiple comparisons test. **P < 0.01, ns = not significant.

The MakA/B/E tripartite on surfaces of red blood cells was visualized using confocal microscopy (Fig. 2*A*). The binding kinetics of the Mak tripartite to a lipid membrane in real time was studied using supported synthetic lipid membrane bilayers of complex composition. The analysis was performed in a quartz crystal microbalance with dissipation monitoring (QCM-D) of the protein adsorption (*SI Appendix*, Fig. S3*B*), confirming binding to the bilayer surface. The proteins were stably bound and were not washed off by rinsing.

Transmission electron microscopy (TEM) was used to investigate if the MakA/B/E tripartite or the individual proteins would form recognizable oligomeric or pore-like assemblies on liposomes prepared from *E. coli* total lipid extracts. Assemblies were indeed observed with the MakA/B/E tripartite (Fig. 2*B*) and among the individual Mak proteins; only MakA formed seemingly well-organized star-shaped oligomers.

We used human colon cancer cells (Caco-2 cells) to investigate if Alexa568-labeled Mak proteins would bind to epithelial cells. Caco-2 cells are human colon adenocarcinoma cells isolated from a primary colonic tumor (24). Flow cytometry was used to monitor binding and/or uptake. The results indicated that a distinctly higher number of cells were labeled with the MakA/B/E tripartite than with any of the three proteins tested separately (Fig. 2*C*). Of the proteins tested individually, MakA showed higher binding/uptake than MakB or MakE. We also assessed the viability of the Caco-2 cells upon 24-h treatment with the Mak proteins. The MakA/B/E tripartite displayed the most pronounced cytotoxic activity, resulting in a loss of viability in about 80% of the Caco-2 cells (Fig. 2*D*). A lower, but still significant, degree of cytotoxicity was observed with MakA alone.

**Fig. 3.**
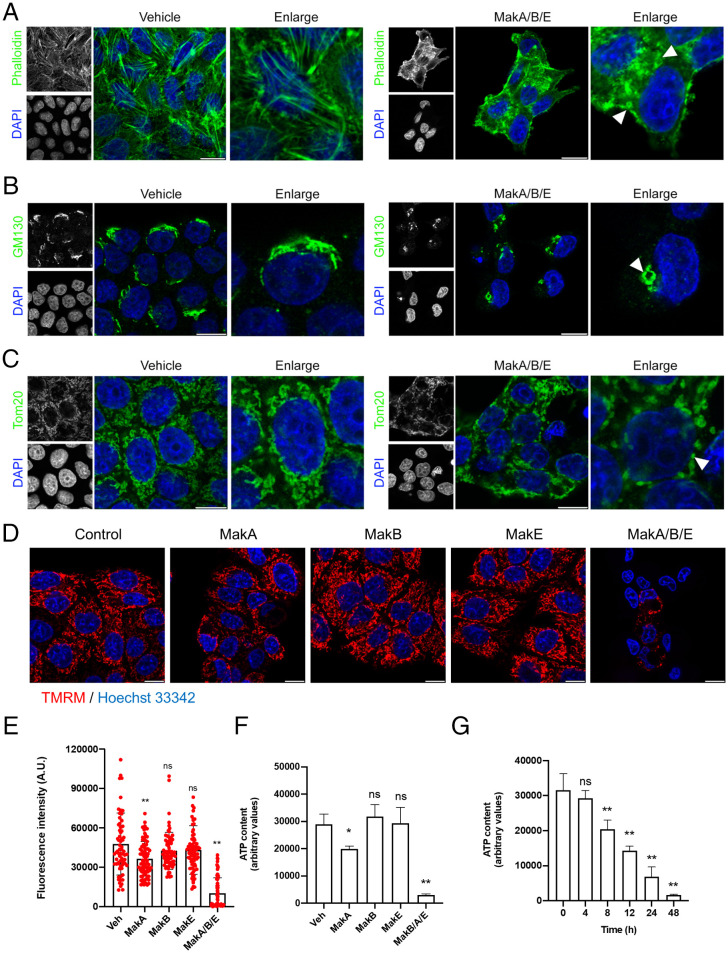
Effect of the MakA/B/E tripartite on intracellular structures and organelles. Caco-2 cells treated with vehicle or MakA/B/E (250 nM, equimolar concentration) for 24 h were examined by confocal laser scanning microscopy. Nuclei were counterstained with DAPI or Hoechst 33342. (Scale bars, 10 µm.) (*A*) Effect on actin filaments. Cells were stained with phalloidin-FITC to visualize actin filaments. Arrowheads (white) indicate disruption of actin filaments. (*B*) Effect on the Golgi apparatus. Cells were stained with a cis-Golgi marker, GM130. Arrowhead (white) indicates changes in the cellular distribution of the Golgi complexes. (*C*) Effect on mitochondria. Immunofluorescence detection was performed with antibodies against Tom20 to visualize mitochondria. Arrowhead (white) indicates swelling of mitochondria. (*D*) Caco-2 cells treated with vehicle, individual components of the tripartite complex, or MakA/B/E (24 h) and stained with mitochondrial potential marker, TMRM (250 nM, 30 min). (Scale bars, 10 µm.) (*E*) Quantification of fluorescence intensity displayed upon treatment of Caco-2 cells as shown in *D*. Bar graphs show mean ± SEM. Data points represent fluorescence intensity of 68 to 81 individual cells. (*F*) Effect on cellular ATP content. Caco-2 cells were treated with 250 nM MakA, MakB, and MakE proteins individually and the tripartite combination at equimolar concentration for 48 h. Histogram represents data from four biologically independent experiments. Bar graphs show mean ± SD. (*G*) Caco-2 cells were treated with an equimolar concentration of the MakA/B/E tripartite (250 nM) in a time-dependent manner. Histogram represents data from three independent experiments. Bar graphs show mean ± SD. In *E* and *F*, the significance was determined from replicates using one-way ANOVA with Dunnett’s multiple comparisons test. *P < 0.05, **P < 0.01, ns = not significant.

To determine if the MakA/B/E tripartite disrupted intracellular structures of epithelial cells, Caco-2 cells were stained for 1) actin filaments using phalloidin-fluorescein isothiocyanate (FITC), 2) the cis-Golgi marker, GM130, or 3) the mitochondrial marker, Tom20. The tripartite combination induced distinct changes in the cellular distribution of the three markers (Fig. 3 *A–C*). Importantly, the MakA/B/E tripartite disrupted the actin filaments (Fig. 3*A*) and induced Golgi fragmentation (Fig. 3*B*). It also caused rounding of mitochondria, as evidenced by redistribution of Tom20 staining from filamentous mitochondria to round structures (Fig. 3*C*). MakB or MakE failed to induce any detectable change of the cellular distribution of the actin, Golgi or mitochondrial markers, whereas MakA had a weak effect on Golgi and mitochondria (*SI Appendix*, Fig. S4 *A–C*). Results with Caco-2 cells stained for the mitochondrial potential marker tetramethylrhodamine methyl ester (TMRM) indicated that the MakA/B/E tripartite caused depolarization of mitochondria (Fig. 3 *D* and *E*). The individual components had very little to no effect on the mitochondrial potential (Fig. 3 *D* and *E*). Similar results were obtained with HCT8 cells (*SI Appendix*, Fig. S4 *D* and *E*) that are human adenocarcinoma cells from the ileocecal region (25). The effect on total cellular adenosine triphosphate (ATP) was measured in Caco-2 cells and showed that the tripartite caused the most severe ATP depletion. (Fig. 3*F*). Importantly, the tripartite caused a time-dependent decrease in total cellular ATP content (Fig. 3*G*). Together, these results suggest that the MakA/B/E tripartite mediated disruption of cell organelles, leading to dysfunction of mitochondria.

### Structures of MakA, MakB, and MakE.

The MakA (Protein Data Bank [PDB] 6EZV) crystal structure provided the first details of a protein encoded by the *mak* operon (11). MakA is organized into two domains, a long tail domain consisting of a five-helix bundle and a head domain comprising shorter helices and a β-sheet (strand order β1β3β2β4). MakB and MakE were predicted to have the same fold as MakA despite the low sequence identity (20% with MakB and 25% with MakE). Selenomethionine (SeMet)-labeled MakB and MakE were prepared and used for single-wavelength anomalous diffraction (SAD) phasing. For both proteins, initial models were built from the SeMet-phased electron density maps and used for further model building using native data. MakB and MakE were refined to 2.1 and 2.0 Å resolution, respectively (*SI Appendix*, Table S1).

**Fig. 4.**
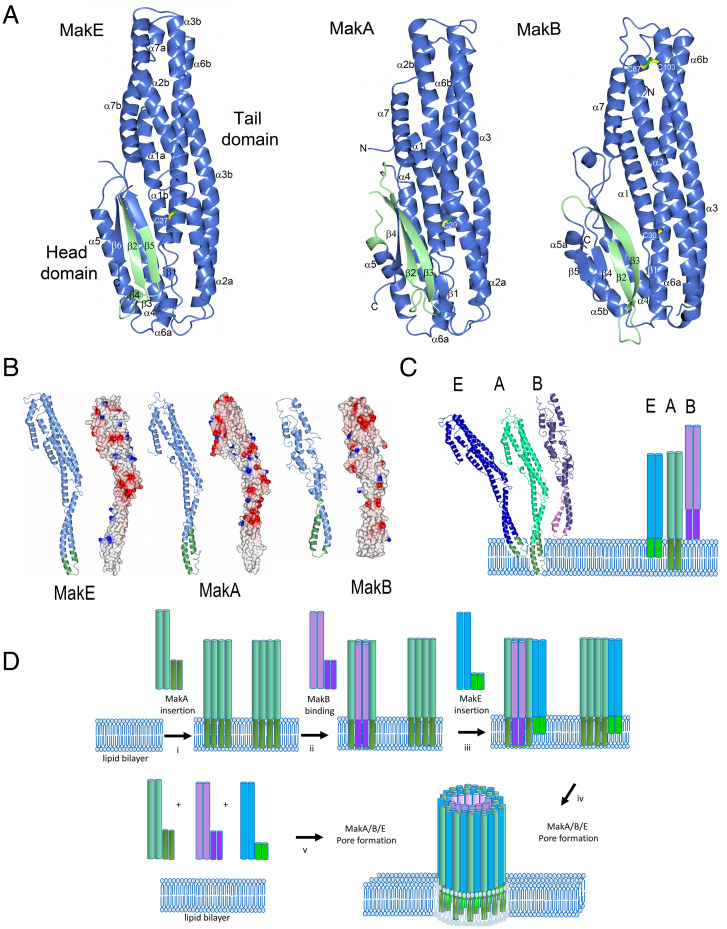
The structures of MakE, MakA, and MakB. (*A*) The crystal structures of MakE, MakA, and MakB proteins are presented as ribbon diagrams, depicted in blue with the hydrophobic β-tongue in green. The conserved cysteine (located on the first helix of all MakA/B/E proteins) and the disulfide in MakB are indicated as stick models. The head and tail domains are labeled. (*B*) Elongated models of MakE, MakA, and MakB, calculated from the AhlB and AhlC structures (18). The region originating from the β-tongue is shown in green. Their electrostatic properties are shown in the same orientation. (*C*) A schematic illustration of how the Mak proteins may interact with a membrane. Their hydrophobic regions are shown in green (MakE and MakA) and pink (MakB). The results from pull-down experiments with liposomes indicated that both MakA and MakE could bind to membranes independent of any other component, whereas MakB seemed to require a partner component (*SI Appendix*, Fig. S10). The two transmembrane helices in MakA would presumably allow for the most efficient anchoring on the membrane of the Mak proteins. The hydrophobic region of MakE is shorter and can only traverse one leaflet of the membrane, while it also could bind independently. MakB seemed unable to bind the membrane stably by itself and appeared to form a complex primarily with MakA. (*D*) A schematic illustration of the two most efficient cases of how pore formation may occur as suggested by results from hemolysis assays (Fig. 1 *E* and *F*). Sequential addition of the MakA, MakB and MakE proteins: (*i*) MakA insertion as multimers (dimers, trimers, etc.) and (*ii*) MakB interaction with MakA before (*iii*) MakE insertion occurs with binding to the MakA–MakB complex, which results (*iv*) in efficient pore formation. Simultaneous addition of a preformed equimolar mixture of the MakA, MakB, and MakE proteins: (*v*) The Mak protein mixture resulted in efficient pore formation when presented to the erythrocyte membrane.

MakE is structurally very similar to MakA, with a root mean square deviation of 2.3 Å calculated on 329 aligned Cα atoms. Its tail domain, 85 Å in length, consists of five long helices (α1, α2, α3, α6, and α7). The head domain, 55 Å in length, comprises two parallel helices (α4 and α5) and a four-stranded antiparallel β-sheet (strand order β1β5β2β6). β1 is very short and originates from the tail domain. MakE also has a β-hairpin (β3β4) protruding 90° from the sheet. (Fig. 4*A*). Two molecules of MakE are found in the asymmetric unit, along with two nickel ions and four sulfate ions.

MakB has the same overall fold as MakA and MakE but deviates more from MakA, with an r.m.s.d of 3.0 Å calculated on 328 Cα atoms. The MakB tail domain is very similar to that of MakA; differences are mainly found in the head domain in which MakB comprises a larger β-sheet (strand order β1β3β2β4β5) than MakA. One MakB molecule is found in the asymmetric unit in which we also modeled two sulfate ions. A comparison of MakA, MakB, and MakE revealed the similarity of their orientation and tight packing between the head and tail domains. In the tail domains, all three proteins have a bend in the α2-helix in equivalent positions. In MakA and MakB, the bend is formed in close proximity to a proline (Pro70 and Pro67, respectively). MakE, which has the most prominent break of the α2-helix, has no proline at this position. All three proteins have a conserved cysteine positioned in the tail domain. In MakA and MakE, the cysteine is a few residues from the end of α1 (Cys29 and Cys27, respectively), and in MakB, it is the final residue of α1 (Cys30) (Fig. 4*A*). MakB has two additional cysteines, Cys87 and Cys103, which form a disulfide bond that rigidifies the connection between the carboxyl terminus of α2 with the N terminus of α3. The head domains share the same overall fold but differ in the number of β-strands and in the lengths of the loops. Except for the cysteines in α1, sequence alignment revealed very few conserved residues. (*SI Appendix*, Fig. S5).

### Predicted Transmembrane Helices in MakA, MakB, and MakE.

The program TMHMM (26) predicted transmembrane helices, or a hydrophobic region, in the head domains of MakA, MakB, and MakE (*SI Appendix*, Figs. S5 and S6). In MakA, the residues 198 through 246 were predicted as two transmembrane helices, and in MakE, one transmembrane helix was predicted from residues 202 through 224. In MakB, the program detected a stretch with increased hydrophobicity, residues 194 through 228; however, a transmembrane helix was not suggested. When these regions were mapped onto the respective crystal structures, it was clear that they built up the two central β-strands and the beginning of α5 of MakA. In contrast, the hydrophobic region in MakE constitutes the first central β-strand (β2), the β-hairpin β3β4, and half of the next β-strand (β5). In MakB, the hydrophobic region constitutes most of β2 and all of β3 (Fig. 4*A*). Most of these hydrophobic residues are shielded from solvent by packing against α1 of the tail domain and the helices of the head domain. In accordance with the nomenclature used for ClyA, which has a similar hydrophobic stretch, we referred to this region as the “β-tongue.”

Analyses of MakA, MakB, and MakE with the protein structure comparison server DALI (27) identified a number of structurally related proteins. The top hits with Z-scores of 20 or higher were AhlB, SmhA, and SmhB (PDB 6GRK, 7A27, and 6ZZ5) from *A. hydrophila* and *S. marcescens* (18, 19), followed by the two toxin components from *B. cereus,* HBL-B and NheA (PDB 2NRJ and 4K1P) (28, 29) and the nematicidal toxin Cry6A (PDB 5GHE) from *Bacillus thuringiensis* (30). Notably, Ahl, Smh, HBL, and Nhe have been described as tripartite toxins (17–19). Further down the list (Z-score 18) was an elongated form of AhlB (PDB 6GRJ) followed by the bicomponent toxins YaxA, XaxA, and PaxB with Z-scores of 11 through 13 (PDB 6EK7, 6GY8, and 6EK4) (31, 32). A protomer form of ClyA (PDB 2WCD) (33) was found with a Z-score of 11. In addition, we performed protein homology searches for MakA, MakB, and MakE using the Phyre2 database (34). The structurally related bacterial toxins identified were analyzed for a phylogenetic relationship that revealed clustering of MakA, MakB, and MakE together with NheA (*SI Appendix*, Fig. S7). Most of these proteins had a hydrophobic cluster ranging from 150 to 300 amino acid residues, except XaxB and YaxB (*SI Appendix*, Fig. S6).

The food poisoning bacterium *B. cereus* expresses at least two tripartite toxins, HBL and Nhe (17). HBL consists of HBL-B, HBL-L1, and HBL-L2 and the Nhe enterotoxin of NheA, NheB, and NheC. As described in the structure comparison section, the crystal structures of HBL-B and NheA have been determined (28, 29) and are structurally very similar to MakA, MakB, and MakE. HBL-L1, HBL-L2, NheB, and NheC are predicted to have similar folds.

Recently, crystal structures of the *A. hydrophila* proteins AhlB and AhlC, components of the tripartite AhlA/B/C toxin, and of the *S. marcescens* proteins SmhA and SmhB, components of the tripartite SmhA/B/C toxin, were reported (18, 19). The respective third components, AhlA and SmhC, were predicted to be structurally similar to the other Ahl and Smh proteins. Most of these proteins have a predicted hydrophobic β-tongue located in an equivalent position as in MakA, MakB, and MakE (Fig. 4*A*). Interestingly, in each of the currently described tripartite toxins (Mak, HBL, Nhe, Ahl, and Smh), one of the components (MakA, HBL-L1, NheB, AhlB, or SmhB) exhibits a hydrophobic β-tongue equivalent to two transmembrane helices. A shorter β-tongue, equivalent to one transmembrane helix, is found in MakE, NheC, AhlC, and SmhC, whereas less-pronounced hydrophobic regions are found in MakB and HBL-B. In NheA, HBL-L2, AhlA, and SmhA, the TMHMHH program did not identify any significant hydrophobic regions.

The Ahl structures were determined in alternative crystal forms, which allowed the identification of two major folds. One AhlB fold is very similar to that seen in our crystal structures of MakA, MakB, and MakE (thus the high Z-score). Like the Mak proteins, AhlB has the same break in α2 due to the presence of a proline and a cysteine located at the end of α1 (*SI Appendix*, Fig. S8). In the other AhlB and AhlC forms, the head domain is rearranged into a helical extension of the tail helices, resulting in a 150-Å-long protein. This form is described as the elongated pore form, and the hydrophobic β-tongue is now found at the end of α3 and the beginning of α4. We used the pore forms of AhlB and AhlC (PDB 6GRJ and 6H2E) to model elongated forms of MakA, MakB, and MakE (Fig. 4*B*). As a result, we obtained a model of MakA with two hydrophobic regions at the cusp of α3 and α4, long enough to span over two leaflets of a membrane (38 Å). Similarly, an elongated form of MakE was modeled in which the tip of the long α3/α4 helices is hydrophobic, albeit the hydrophobic residues are more concentrated on one side. The pore form of AhlC was used to model MakB and generated a structure in which the tip of the protein has one more hydrophobic side and one more polar side (Fig. 4*B*).

### Formation of MakA/B/E Tripartite Oligomers.

MakA, MakB, and MakE acted as monomers during gel filtration and eluted at 15.6 mL from a Superdex200 10/300 column. When MakA, MakB, and MakE were mixed with the detergents n-Dodecyl β-D-maltoside or cymal5 in the presence of lipid extracts (LE), an elution peak at 13.0 mL was obtained, indicating formation of an oligomeric complex. However, when incubated with lauryl maltose neopentyl glycol and LE, the elution volume shifted to 12.1 mL, which indicated formation of a higher oligomeric complex. No oligomers were detected of MakA, MakB, or MakE in bipartite combinations (*SI Appendix*, Fig. S9).

In the study of the Ahl toxin, it was shown that a triple leucine to threonine mutation in the AhlC transmembrane region reduced the hemolytic activity of the AhlA/B/C tripartite (18). We tested the effect of similar mutations in MakA. *E. coli* with plasmids expressing the wild-type *makB^+^A^+^E^+^* genes or constructs with mutations in the *makA* gene were used in vivo in the *C. elegans* killing assay. In comparison with the wild-type construct, the *E. coli* derivative harboring p*makB^+^A_L193T,L246T_E*^+^ showed reduced toxicity toward *C. elegans*. Furthermore, the *E. coli* derivative harboring p*makB^+^A_L193T,L207T,L213T,L246T_E*^+^ appeared completely attenuated in its toxicity toward *C. elegans* (Fig. 1*C*). Compared to the wild-type, the mutants expressed the same level of all Mak proteins. However, while the secretion of MakB and MakE remained unaffected in the mutants, secretion of MakA was reduced (Fig. 1*D*). These results show that the hydrophobic head domain of MakA plays an important role for its secretion and, subsequently, for the toxicity toward *C. elegans*.

Consistent with the prediction by the DALI server (27), our results suggest that MakA, MakB, and MakE most likely form a tripartite pore in membranes in a manner resembling the structures described for AhlA/B/C from *A. hydrophila* (18). To further investigate the possible mechanism of pore assembly of the MakA/B/E tripartite in the presence of lipid membranes, we performed liposome pull-down experiments. In short, liposomes were incubated with the MakA/B/E tripartite mixture or with individual MakA, MakB, or MakE, followed by cross-linking and centrifugation to pull-down cross-linked liposome–protein complexes (*SI Appendix*, Fig. S10). When incubated with MakA/B/E, several oligomeric forms were detected with the MakA antiserum. The most pronounced were monomers and dimers. Incubation with MakA alone resulted in a similar pattern. With the MakB and MakE antisera, mainly larger oligomers were detected but also dimers and tetramers (MakE) and trimers (MakB). Incubation with MakE alone resulted in monomers only, whereas with MakB alone, no protein was detected, suggesting that MakB was not efficiently binding to the liposomes used in the pull-down assay (*SI Appendix*, Fig. S10). We conclude that MakA could bind to liposomes independently of other proteins and that it formed dimers, tetramers, and larger oligomers. It appeared that MakE bound rather weakly to liposomes and did not oligomerize. In the case of MakB alone, we did not detect any binding. (*SI Appendix*, Fig. S10). These findings indicated that binding of MakA to the liposomes facilitated interaction and subsequent oligomerization of MakB and MakE together with MakA. On the basis of the results from these pull-down experiments and from the hemolysis assays with different sequential orders and combinations of Mak proteins (Fig. 1 *E* and *F*), we propose a model for the MakA/B/E tripartite lytic membrane pore formation (Fig. 4 *C* and *D*). In short, we assume that MakA, MakB, and MakE are released from the bacteria via the *Vibrio* flagellum in their elongated forms, in which the head domains have rearranged into two long parallel helices. In MakA, the hydrophobic β-tongue is located at the ends of these helices and can penetrate both leaflets of the host membrane, anchoring MakA firmly. Next, MakB binds to the membrane-anchored MakA and arranges itself as a scaffold for the pore-like complex. Finally, MakE joins the assembly and interacts with MakA and/or MakB. Additionally, its hydrophobic region penetrates one leaflet of the membrane.

### Phylogenetic Distribution of the *mak* Gene Cluster among Vibrionaceae Strains.

**Fig. 5.**
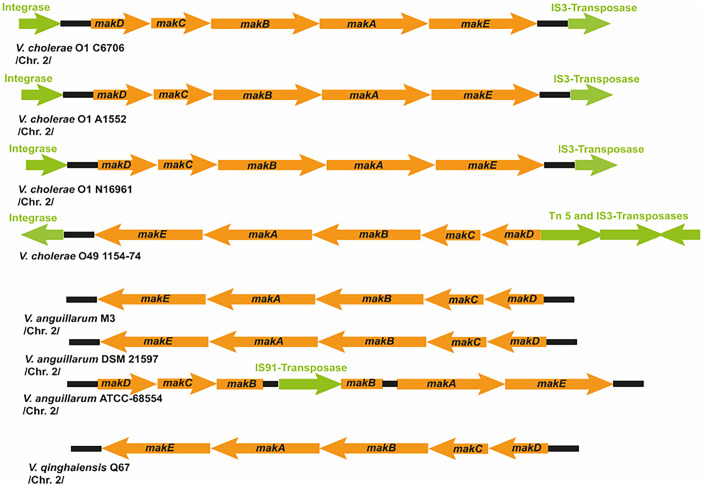
Presence of the *mak* gene cluster in different Vibrionaceae strains. Gene organization of *mak* gene clusters in the genomes of representative *V. cholerae*, *V. anguillarum*, and *V. qinghalensis* strains. Genetic information of transposable elements in flanking regions and within the gene clusters are indicated in green.

To further investigate the biological relevance of Mak proteins, we focused on their genetic determinants organized as the discrete gene cluster *makDCBAE*, found in *V. cholerae* strain A1552 (Fig. 1*A*). Bioinformatics analyses of over 100 sequenced genomes of *Vibrio* spp. revealed that the complete *mak* gene cluster is specifically distributed among distinct strains of *V. cholerae, V. anguillarum*, and *Vibrio qinghaiensis* (*SI Appendix*, Table S2). The strains include different serogroups (O1, O139, and non-O1/O139) and both the classical and the El Tor biovars. Among them are representatives of several of the proposed six stages in the evolution of the current seventh pandemic of *V. cholerae* (35). The gene cluster was predominantly located on the second chromosome (Chr. 2) and was present in *V. cholerae* with a single chromosome (Fig. 5 and *SI Appendix*, Table S2). A few species-specific characteristics also emerged. In *V. cholerae*, the *mak* gene cluster is predominantly located in the clockwise orientation of their chromosomes, flanked by nearby genetic elements putatively coding for activities implicated in lateral gene transfer (LGT): integrases and transposases (Fig. 5 and *SI Appendix*, Table S2). The *mak* genes found in *V. anguillarum* and *V. qinghaiensis* are mostly present in the counterclockwise orientation and are not immediately bordered by LGT-promoting elements (Fig. 5 and *SI Appendix*, Table S2).

These findings, together with the estimation that *V. anguillarum* emerged earlier in evolution than *V. cholerae* (36), made us explore the phylogenetic distribution of the *mak* gene cluster among the Vibrionaceae strains listed in *SI Appendix*, Table S2. We constructed a phylogenetic tree using the *mak* gene cluster of *V. cholerae* with the flanking LGT-promoting genes (*SI Appendix*, Fig. S11). The tree depicted two main groups: one composed of strains of *V. anguillarum* and *V. qinghaiensis* and the other group by strains of *V. cholerae* (*SI Appendix*, Fig. S11*A*). The calculated evolutionary distance suggested that the *mak* operon was acquired earlier and then preserved by *V. anguillarum*, consistent with the lack of flanking LGT genes (Fig. 5 and *SI Appendix*, Fig. S11*B*). In *V. cholerae*, the *mak* genes may have been acquired more recently. In addition to putative transposase and integrase genes, the lower guanine–cytosine (GC) content (41%) compared to the overall genomic GC content (47.5%) is consistent with the idea that the *mak* operon of *V. cholerae* strains may be an acquired genomic island.

## Discussion

Our identification and structural characterization of MakA (11) prompted this investigation of the other proteins encoded by the gene cluster. Here, we establish that MakA, MakB, and MakE together can form a tripartite cytolytic toxin. The *V. cholerae makDCBAE* operon is positively regulated by HapR, a transcriptional regulator of quorum sensing that also represses CT expression (11). In addition, transcription of the *makDCBAE* operon is growth phase dependent; it is specifically activated by the transcriptional regulator RpoS during stationary growth phase (37). The Mak proteins are thereby expected to be less expressed during acute cholera disease. Instead, in natural aquatic environments where nutrition and predation constraints challenge bacterial fitness and survival, they are more likely produced. This is the first described tripartite cytotoxin from *V. cholerae*, and our findings are important for understanding its possible involvement in bacterial fitness and pathogenesis during infection in a variety of hosts and environments.

For structural analyses, MakA, MakB, and MakE were crystallized in their soluble, compact forms in which the hydrophobic β-tongue is hidden in the interface between their respective head and tail domain. This region is longer in MakA (48 residues) than in MakE (22 residues), whereas the equivalent region in MakB is amphipathic. Each protein is predicted to undergo a conformational change in which the head domain is unfolded and rearranged into two long helices in order to assemble into a membrane-bound tripartite complex (Fig. 1 *C* and *D*). Such a model was discussed for the tripartite Ahl and Sml toxins (18, 19) and was comprehensively addressed for the pore-forming toxin ClyA (38). Based on this proposed theory, the tripartite assembly of MakA/B/E can be postulated as follows. In MakA, the hydrophobic β-tongue is long enough to traverse the membrane bilayer. MakE, with its shorter β-tongue, can penetrate only a single membrane leaflet. On the other hand, because MakB is less hydrophobic, it is considered to not bind the membrane directly. Consequently, one can expect that MakA anchors to the membrane due to its more pronounced hydrophobicity and thereafter attracts MakB and MakE to the complex (Fig. 1*D*).

The assembly pathway for the tripartite complex was further supported by the observations when MakA, MakB, and MakE sequentially were introduced to erythrocytes in different order. When all proteins were added together as a preformed equimolar mixture or in the order MakA → MakB → MakE in 15-min intervals, the level of hemolysis was as high as in the addition of the positive control, the pore-forming toxin ClyA (Fig. 1*E*). When MakA and MakB were combined in a preformed equimolar mixture, followed by the addition of MakE, hemolysis also occurred, although at a slightly lower level. We hypothesize that the first step, the conformational shift and subsequent membrane attachment of MakA, is the most critical. Both unbound and membrane-bound MakA appear to attract MakB, which would undergo a similar conformational change and interact with MakA but not directly with the membrane. Formation of a pore complex required that MakE, after the structural rearrangement, joined with the MakA/B complex and with the outer leaflet of the membrane via the hydrophobic helices (Fig. 4*D*). Erythrocytes treated with MakB and/or MakE before adding MakA in all other tested combinations resulted in little or no hemolysis, suggesting that insertion of MakE into a MakA–MakB–membrane complex might be the final step that promotes efficient formation of a pore.

MakC and MakD have primary structures and sizes different from the MakA/B/E proteins, and we conclude that they are not essential for Mak cytotoxicity per se. The proposed role as accessory proteins that function during MakA/B/E toxin biogenesis/secretion is yet unknown.

In addition to the hemolytic activity and the toxicity to *C. elegans*, we investigated the effect of MakA/B/E on colon carcinoma cells. Some cytotoxicity could be detected by treatment with MakA alone, probably due to its capacity to bind the membrane. The MakA/B/E combination was considerably more cytotoxic and resulted in destabilization of actin filaments and destruction of mitochondria and the Golgi apparatus. Taken together, these studies provided evidence that the tripartite MakA/B/E complex has the capability to cause such effects if presented to host cells. However, it is questionable if these observations provide a valid reason to assume that, for example, mitochondrial disruption is a terminal, or even intended, outcome of MakA/B/E activity. Our in vitro results merely show that this may be the outcome if MakA/B/E is introduced to mammalian cells.

Recent studies with MakA have revealed that it also can act as a toxin on its own and upon internalization may cause apoptotic responses and autophagy in cultured mammalian cells (39–41). It is an interesting possibility that MakA can play a dual role as a standalone toxin and as a part of a tripartite toxin, as shown in the present study. Notably, some MakA secretion was observed from the flagellum-deficient bacteria, and the three Mak proteins appeared not to be dependent on each other, per se, for secretion. However, efficient secretion of all three Mak proteins was shown to be flagellum dependent, and the *mak* operon structure strongly indicates that these Mak proteins evolved to form a tripartite complex. The fact that *V. cholerae* typically has just one polar flagellum is particularly interesting in terms of Mak toxin secretion. The single flagellum would enable spatially coordinated secretion of the three toxin subunits to the target membrane.

In addition to their structural similarities among the five different bacterial tripartite toxins that have been described so far, there are interesting questions to be clarified regarding their biogenesis. HBL and Nhe are expressed by the Gram-positive *B. cereus*, and Ahl, Smh, and Mak are expressed by the Gram-negative bacteria *A. hydrophila*, *S. marcescens*, and *V. cholerae*, respectively. The HBL and Nhe proteins are produced with N-terminal signal sequences indicating that they are transported over the membrane via the Sec pathway (42). Apart from the information that secretion of the HBL protein requires functional flagella (43), the actual mechanism of secretion remains to be clarified, and it is feasible to consider a route involving the flagella as shown for MakA. The Nhe proteins are presumably exported via an alternative mechanism that is not yet identified (44). It is not known how the Smh and Ahl proteins are exported, but *S. marcescens* has flagella [e.g., fT3SS (45)], and *A. hydrophila* has both a vT3SS secretion system and flagella (46).

While the Mak proteins presumably play an important role for the fitness and survival of vibrios in different hosts, the *makDCBAE* operon on Chr. 2 may also be viewed as an example of how an operon may evolve differently in species that have faced similar challenges. The similarities, such as LGT genes flanking the operon, the low GC content, and the presence of the degenerated *attB* site sequences upstream of the promoter proximal gene (*makD*) that are shared with the *V. cholerae* pathogenicity islands VP1 (47), VSP-1, and VSP-2 (48) support the idea that the *makDCBAE* operon represents a chromosomal genomic island. While the *makDCBAE* operon of *V. cholerae* may be in the process of assimilation, as evidenced by the remnants of the LGT genes, the *makDCBAE* operon of *V. anguillarum* seems to be fully assimilated on Chr. 2. Our present findings will also be of particular relevance for further understanding of *V. anguillarium* virulence and pathology during disease in fish, which is causing high mortalities and economic losses in aquaculture. We envision that the *mak* genes and proteins may be explored as potential targets for new diagnostics and therapeutic strategies, which are in great demand toward different pathogenic Vibrionaceae.

## Materials and Methods

The full experimental procedures are provided in *SI Appendix*, *Materials and Methods*. Briefly, analyses of protein expression, secretion, and complementation experiments were previously described (11). *V. cholerae* O1 El Tor strain A1552 and mutants were prepared as reported (11, 49). Sodium dodecyl sulphate-polyacrylamide gel electrophoresis (SDS-PAGE) and immunoblot analysis were performed according to standard procedures (50). Mak proteins were detected with appropriate antisera. Recombinant protein was prepared as reported previously with some modifications (11) and crystallized by sitting drop vapor diffusion. The structures were solved by SAD. The Mak proteins were analyzed individually and in bipartite and tripartite combinations with gel filtration using buffers with and without detergents and LE. On horse blood agar plates and in human erythrocyte suspensions, hemolytic activity was observed. The viability of Caco-2 and HCT8 cells after treatment with Mak proteins was assessed by a decrease in MTS (3-(4,5-dimethylthiazol-2-yl)-5-(3-carboxymethoxyphenyl)-2-(4-sulfophenyl)-2H-tetrazolium) absorbance. Cellular ATP content was measured with an ATPLite kit (PerkinElmer) on an Infinite M200 microplate reader (Tecan). Using a Leica SP8 inverted confocal system, immunofluorescence was detected using Alexa488/555-labeled secondary antibodies. The uptake of Alexa568-MakA, Alexa568-MakB, Alexa568-MakE, or Alexa568-MakA/B/E by Caco-2 was examined using flow cytometry (BD Accuri C6). Liposomes were prepared using the lipid film hydration and extrusion method. QCM-D, pull-down assays, and electron microscopy were used to investigate the interaction between Mak proteins and liposomes. The maximum likelihood method was used to infer evolutionary connections based on *mak* gene clusters in *Vibrio* genomes.

## Supplementary Material

Supplementary File

## Data Availability

Crystallographic coordinates and structure factors have been deposited in the PDB under accession codes 6TAO (MakE) and 6T8D (MakB).
