# Supplementary material for: A tripartite cytolytic toxin formed by Vibrio cholerae proteins with flagellum-facilitated secretion
Source: Proc Natl Acad Sci U S A. 2021 Nov 19;118(47):e2111418118. doi: 10.1073/pnas.2111418118 (PMC8617504; doi:10.1073/pnas.2111418118)
Supplement: Supplementary File [file pnas.2111418118.sapp.pdf]

Supplementary information

A tripartite cytolytic toxin formed by *Vibrio cholerae* proteins with flagellum-facilitated secretion

Aftab Nadeem[a,b,c,1], Raghavendra Nagampalli[c,d,1], Eric Toh[a,b,c], Athar Alam[c,e], Si Lhyam Myint[a,b,c], Thomas V. Heidler[c,d], Mitesh Dongre[a,b,c], Nikola Zlatkov[a,b,c], Hudson Pace[c,e,f], Fouzia Bano[c,e,f], Anders Sjöstedt[c,e], Marta Bally[c,e,f], Bernt Eric Uhlin[a,b,c], Sun Nyunt Wai[a,b,c,2] and Karina Persson[c,d,2]

[a]Department of Molecular Biology, Umeå University, SE-90187 Umeå, Sweden

[b]Laboratory for Molecular Infection Medicine Sweden (MIMS), Umeå University, SE-90187 Umeå, Sweden.

[c]Umeå Centre for Microbial Research (UCMR), Umeå University, SE-90187 Umeå, Sweden.

[d]Department of Chemistry, Umeå University, SE-90187, Umeå, Sweden.

[e]Department of Clinical Microbiology, Umeå University, SE-90185, Umeå, Sweden.

[f]Wallenberg Centre for Molecular Medicine, Umeå University, SE-90185, Umeå, Sweden.

[1]These authors contributed equally

[2]Corresponding authors: Sun Nyunt Wai
E-mail: sun.nyunt.wai@umu.se
Tel: +46-90-7856704

Karina Persson
E-mail: karina.persson@umu.se
Tel: +46-90-7865926

Supplementary information

Contents:

Materials and Methods
Supporting Figures 1-11
Supporting Tables 1-4
References

Materials and Methods:

Bacterial strains and plasmids

All bacterial strains and plasmids used in this study are listed in **Supplementary Table 3**.

Vibrio cholerae strain mutagenesis and growth conditions

The *Vibrio cholerae* strains used for this study were the wild-type *V. cholerae* O1 El Tor strain A1552 and derivatives with the following mutations described earlier: Δ*makA*, Δ*makB*, Δ*makC*, Δ*makD* and Δ*flhA,* respectively (1). For the construction of a Δ*makE* mutant derivative, the oligonucleotide primers used were based on *V. cholerae* O1 El Tor whole genome sequence (2). PCR amplification was performed using Phusion DNA polymerase (Thermo Scientific) and primers presented in Supplementary Table 4. The *makE* deletion in *V. cholerae* was obtained using the procedure with positive selection for allelic exchange as described (3). For analyses of Mak protein expression and secretion, the *V. cholerae* strains were grown in LB media and incubated at 37 °C as previously described (1).

MakB and MakC complementation experiments

The *makB* and *makC* clones in the pBAD18 vector (1) were electroporated into Δ*makB* and Δ*makC* mutants, respectively. As a negative control, the expression vectors pBAD18 without an insert was introduced into the mutant strains.

Cloning, overexpression and purification of Mak proteins.

Details about MakA cloning, overexpression and purification were described earlier (1)The *makB* and *makE* genes (UniProt accession codes Q9KL65 and Q9KL63) were PCR amplified from genomic DNA of *V. cholerae* strain A1552. The *makB* PCR product was digested with *NcoI*/*XhoI*, the *makE* with *BspHI/HindIII*. Each product was cloned into equivalent sites of pET-His1a, in-frame with a cleavable histidine affinity tag with the sequence MKHHHHHHPMSDYDIPTTENLYFQGA. The plasmids were introduced by transformation into *E. coli* DH5α and positive colonies were subsequently sequenced. The constructs were expressed in *E. coli* BL21(DE3) in LB supplemented with 50 µg/mL kanamycin. When the cultures reached OD_{600} ~0.6, protein expression was induced with 0.5 mM isopropyl 1-thio-β-

d-galactopyranoside (IPTG) followed by growth for 5 h at 25 °C. Cells were harvested by centrifugation, and the pellets were stored at -80 °C until further use. The cell pellets were resuspended in 50 mM Tris-HCl pH 7.6, 0.3 M NaCl, and 10 mM imidazole (lysis buffer) supplemented with 1% triton-X100 and sonicated on ice. The lysate was centrifuged at 63,000 x *g* for 20 min. The resulting supernatant was passed over a column packed with His60 Ni-resin (Takara). The column was washed with lysis buffer containing 30 mM imidazole after which the protein was eluted with the same buffer containing 0.3 M imidazole. Next, the histidine tag was removed by incubation with 1% (w/w) TEV protease overnight at 4 °C. After buffer exchange to 50 mM Tris-HCl pH 7.6, 0.2 M NaCl the protein solution was passed over the affinity column again. The flowthrough and wash fractions were collected and concentrated. The cleaved protein was further purified on a HiLoadTM 16/60 Superdex™200 prep-grade column (GE Healthcare) equilibrated with 20 mM Tris-HCl pH 7.6, 0.2 M NaCl. Fractions containing the peak of interest were concentrated in 20 mM Tris-HCl pH 7.6.

Selenomethionine (SeMet)-labelled MakB and MakE were produced by growing the cultures in M9 media supplemented with glucose at 37 °C. At OD600 ~0.4, 100 mg/L each of lysine, threonine, phenylalanine and 50 mg/L each of leucine, isoleucine, valine, proline and SeMet were added and protein expression was induced with 0.5 mM IPTG (4) at 20 °C overnight. The SeMet-labelled proteins were purified as described above.

Crystallization and structure determination

Sitting-drop vapor-diffusion trials were performed at 20 °C for MakB (8.3 mg/mL) and MakE (20 mg/mL) in 96-well MRC-crystallization plates (Molecular Dimensions). Droplets of 0.5 µL protein were mixed with equal volumes of screening solutions from Molecular Dimensions. For native MakB, the final optimized crystallization condition was 1.5 M $MgSO_4$ and 0.1 M MES pH 6.5. Crystals of SeMet-labelled MakB were obtained in 0.6 M ammonium sulphate, 0.1 M sodium acetate pH 4.5. Native MakE was crystallized in 10 mM $NiCl_2$, 0.1 M Tris-HCl pH 8.5 and 20% (w/v) monomethyl ether polyethylene glycol (PEGmme) 2000. SeMet-labelled MakE was crystallized in 24% (w/v) PEG 3350, 25 mM Na/K phosphate, 0.1 M BisTris propane pH 6.5.

All crystals were soaked for 30 seconds in mother liquor solution supplemented with 20% (v/v) ethylene glycol or 20% (v/v) PEG 400 before they were flash cooled in liquid nitrogen and stored until data collection.

MakB native diffraction data were collected on a Pilatus3 2M detector at beamline ID23-2 and single anomalous diffraction (SAD) data at beamline ID23-1 on a Pilatus 6M F detector at the

Supplementary information

European Synchrotron Radiation Facility, Grenoble, France (ESRF). Native MakE data were collected at the ESRF beamline ID23-2 on a Pilatus3 2M detector. SAD data were collected at beamline BioMAX, MAX IV in Lund, Sweden. Diffraction images were processed with XDS (5) and scaled with Aimless (6) from the CCP4 program suite (7). The structure of SeMet-labelled MakB was solved with SAD-phasing using AutoRickshaw (8). Density modification and automatic model building were performed using AutoRickshaw and ArpWarp (9) and refined using phenix.refine (10). The SAD MakE data were carefully reprocessed using XDS (5), after which the scaled data were used as input in the CRANK2 pipeline (11). A search for ten Se atoms resulted in an initial model that was further built in COOT (12).

The crystals of the native MakE protein grew in space groups different from the SeMet-labelled proteins, and hence the structure was obtained by molecular replacement using Molrep (13, 14) with the SeMet structure as search model. The native structures were refined using phenix.refine (10) and built using rounds of manual building in COOT (12). For the refinement of MakE, translational-libration-screw refinement was used, treating each molecule as an individual TLS group (15).

Figures were prepared with CCP4mg (16). Relevant processing and refinement statistics are summarized in Supplementary Table1. Elongated models of MakE, MakA, and MakB were obtained with Swissmodel (17) using the structures of AhlB (PDB 6GRJ) and AhlC (PDB 6H2D) as starting models(18).

Analysis of MakA/B/E tripartite and bipartite oligomers using gel filtration

The MakA/B/E tripartite were prepared by incubating equimolar concentrations (250 nM) of MakA, MakB and MakE with 0.1% detergent (Antrace) and lipid extracts (LE; Avanti Polar Lipids) at 25 °C for 1 h. Subsequently, aggregates and MakA/B/E oligomers with high molecular weight were separated by size exclusion chromatography on a Superdex 200 10/300 column (GE Healthcare) in 30 mM MES pH 5.6, 150 mM NaCl, 0.003% detergent and LE buffer. The detergents used were N-dodecyl β-C-maltoside (DDM), Lauryl Maltose Neopentyl Glycol (LMNG) and Cymal5. The bipartite combinations (250 nM) were purified using the same protocol.

Antibodies

For immunodection of Mak proteins by immunofluorescence (IF) or western immunoblot (WB) analyses we used polyclonal antisera from rabbits (produced by GeneCust) at the following dilutions: Anti-MakA (IF= 1:100, WB= 1:5000), Anti-MakB (WB= 1:5000), Anti-MakC (WB= 1:5000), Anti-MakE (WB= 1:5000). Anti-Tom20 (#612278, IF = 1:100) and Anti-GM130

(#610822, IF = 1:100), mouse monoclonal antibodies were purchased from BD Biosciences. Anti-beta-actin mouse monoclonal antibody (#A2228, WB: 1:5000) was purchased from Sigma-Aldrich. Goat anti-Rabbit HRP-conjugated IgG (#AS09602, WB: 1:5000) were purchased from Agrisera AB, Sweden. Rabbit anti-mouse HRP-conjugated immunoglobulins (#P0260, WB: 1:5000) were purchased from Dako. Alexa Fluor 488/555/647 conjugated secondary antibodies for immunofluorescence were purchased from Thermo Fisher.

SDS-PAGE and Immunoblot analysis.

Sodium dodecyl sulphate-polyacrylamide gel electrophoresis (SDS-PAGE) and immunoblot analysis were performed according to standard procedures(19). Briefly, bacterial whole cells and culture supernatants were separated by centrifugation at 10,000 × *g* for 15 min. Cell pellets were suspended in an appropriate volume of 1× SDS buffer and boiled to obtain whole cell lysates. Culture supernatants were filtered through a 0.45 µm PVDF syringe filter (Millipore, USA) and subsequently precipitated with 10% (w/v) trichloroacetic acid (TCA). The samples were centrifuged at 15,000 × *g* for 15 min at 4 °C to pellet the TCA-precipitated proteins, which were then resuspended in 1× SDS buffer and boiled for 5 min. Protein samples were resolved by SDS-PAGE and processed for immunoblotting. HRP-conjugated goat anti-rabbit IgG (Agrisera AB, Sweden) was used as a secondary antibody. Immunoblot detection was done using Clarity Western ECL substrate (BioRad). Prestained protein molecular weight standards (SM0679, Fermentas) were used to determine the protein molecular weights.

Hemolysis assay

For tests of hemolytic activity, Mak proteins (250 nM) were mixed with a suspension of human erythrocytes (2% of whole blood) in Phosphate-Buffered Saline (PBS) and incubated 120 min at 37 °C. The cytolytic *E. coli* protein ClyA (250 nM) and Triton X-100 were used as positive controls and PBS as the negative control. After centrifugation, the supernatants were monitored for released haemoglobin, by measurement of absorbance at 545 nm, as an indicator of red blood cell lysis. ClyA was obtained form from *E. coli* through the procedures for overproduction and purification described earlier (20, 21).

Cell lines and cell culture

Human colon cancer cells (Caco-2; RRID:CVCL_0025 and HCT8; RRID:CVCL_2478) were obtained from American Type Culture Collection (ATCC) and maintained in RPMI-1640 or DMEM media supplemented with non-essential amino acids (1:100), sodium pyruvate (1 mM), penicillin (20 Units), streptomycin (20 µg/mL) and 10% fetal bovine serum (FBS) at 37 °C, 5% CO_2. All experiments were performed with mycoplasma-free cells.

Supplementary information

Cell viability and ATP estimation assays

For cell viability experiments, Caco-2 or HCT8 colon carcinoma cells were grown on a 96-well plate ($5x10^3$/well, Tecan Group Ltd) overnight and treated with the individual Mak proteins for 48 h at 37 °C, 5% CO_2. Loss of cell viability was quantified by a decrease in 3-(4,5-Dimethylthiazol-2-yl)-5-(3-carboxymethoxyphenyl)-2-(4-sulfophenyl)-2H-tetrazolium (MTS; Promega) absorbance. MTS absorbance (490 nm) was measured on an Infinite M200 microplate reader (Tecan) according to the manufacturer's instructions. Data were normalized to an equal volume of vehicle-treated (20 mM Tris-HCl, pH 7.4) cells and expressed as percentages.

For measurement of total cellular adenosine triphosphate (ATP) content, Caco-2 cells were grown on a 96-well plate ($5x10^3$/well, Tecan Group Ltd) overnight and treated with increasing concentrations of the individual Mak proteins or the tripartite complex for 48 h or with the tripartite complex in a time-dependent manner at 37 °C, 5% CO_2. Cellular ATP content was measured with an ATPLite kit (PerkinElmer) on an Infinite M200 microplate reader (Tecan) according to the manufacturer's instructions.

Immunofluorescence

For fixed cell immunofluorescence, Caco-2 cells were grown in a coverslip-bottom 8-well chamber slide (µ-Slide, ibidi) and fixed in 4% paraformaldehyde for 30 min at room temperature (RT), permeabilized in 0.25% Triton X-100 (15 min). Cells were then washed with PBS and incubated with primary antibodies (overnight, 4 °C), followed by incubation with respective Alexa488/555 conjugated secondary antibodies. Nuclei were counterstained with DAPI (5 min, RT). For measurement of the mitochondrial potential, Caco-2 or HCT8 cells were treated with an equal volume of vehicle (20 mM Tris-HCl, pH 7.4), MakA (250 nM), MakB (250 nM), MakE (250 nM) or the tripartite complex (MakA/B/E, 250 nM each) for 24 h. Subsequently, the treated cells were incubated with Image-iT™ Tetramethylrhodamine methyl ester (TMRM) reagent (Thermofisher) for 30 min (250 nM). Cells were visualized using a Leica SP8 inverted confocal system (Leica Microsystems) equipped with a HC PL APO 63x/1.40 oil immersion lens. For quantification of fluorescence intensity, images were captured and processed using the LasX (Leica Microsystems). Images were further processed using ImageJ – FIJI distribution (NIH) (22).

Flow cytometry

Cellular uptake of Alexa568-MakA, Alexa568-MakB, Alexa568-MakE (250 nM, 24 h) or Alexa-MakA/B/E (Equimolar concentration of individual protein, 250 nM, 24 h) treated Caco-2 cells

was investigated by flow cytometry (BD Accuri C6). Live cells were gated, and cellular uptake was represented as mean fluorescence intensity (MFI) (23).

Lipids

All lipids were purchased from Avanti Polar Lipids, Alabaster, AL, USA. Lipids: 1-palmitoyl-2-oleoyl-glycero-3-phosphocholine (POPS), 1,2-dioleoyl-sn-glycero-3-phosphoethanolamine (DOPE), 1-palmitoyl-2-oleoyl-sn-glycero-3-phospho-L-serine (POPS), Sphingomyelin from Porcine Brain (SM), Cholesterol from Ovine (Chol), L-α-phosphatidylinositol-4,5-bisphosphate from Porcine Brain (PIP2), and N-palmitoyl-sphingosine-1-{succinyl[methoxy(polyethylene glycol)5000]} (PEG5Kce). Lyophilized PIP2 lipids were dissolved in a mixture of Chloroform:Methanol (2:1) to a concentration of 1 mg/mL. Next, they were protonated by adding 0.5 µL of 1 M HCl to 100 µg of PIP2, kept at RT for 15 min and dried down with nitrogen gas. The dried lipid was redissolved in a choloform:methanol (3:1) mixture to 1 mg/mL followed by drying again. Finally, the 100 µg of PIP2 was redissolved in 100% chloroform to 1 mg/mL and stored at -20 °C until used for liposome preparation.

Liposome Preparation

Liposomes containing 0.5 mol% PEG5Kce, 5 mol% PIP2, 10 mol% SM, 10 mol% POPS, 15 mol% DOPE, 20 mol% Chol, and 39.5 mol% POPC (referred herein to as synthetic lipid mixture, SLM liposomes) were prepared using the lipid film hydration and extrusion method. The SLM composition was inspired by the distribution of lipids found in the plasma membrane of HeLa cells (24) with slight adjustments made to the mole percentages to improve supported lipid bilayer production. The individual lipids dissolved in chloroform were mixed, dried under nitrogen flow during a minimum of 1 h. The dried lipid film was then rehydrated using a pre-heated citrate buffer at pH 4.5 (20 mM Citrate, 50 mM NaCl, pH 4.5, 37 °C) to a lipid concentration of 1 mg/mL. The solution was then extruded at ~40 °C eleven times through a polycarbonate membrane with 50 nm pore size using an Avanti miniextruder. The liposomes were stored at 4 °C until used.

QCM-D

The QCM-D measurements were carried out using an AWSensors X4 (AWsensors, Spain) instrument equipped with flow chambers and SiO2 coated sensors (Wrapped 14mm, 5 MHz, Cr/Au - SiO2, Polished). Each sensor was stored in 2% SDS overnight and treated with UV-ozone (Bioforce Nanosciences, USA) for 30 min prior to use. Supported lipid bilayers were formed using PM+PEG liposomes (100 µL of 0.1 mg/mL) in citrate buffer at pH 4.5 (20 mM Citrate, 50 mM, 0.1 mM EDTA, pH 4.5) followed by rinsing in the same buffer. The buffer was then exchanged for a buffer containing 120 mM citrate (pH 7), which was also the buffer used

for protein adsorption. MakA, MakB and MakE (17.3 μM each) were mixed together in 120 mM citrate buffer for 10 min before being added. The flow was stopped once the protein reached the chamber, and the bilayer was incubated under steady conditions for 80 min before rinsing with the same buffer. The whole measurement was carried out at 37 °C. The protein layer was found to be rigid (D~0.3*10^{-6}). The frequency shift of the third overtone was therefore converted into a bound mass using the Saurbrey equation.

Liposome pull-down assay

E. coli total lipid extract (Avanti Polar Lipids) dissolved in chloroform was dried to a thin film under a nitrogen stream. The lipid film was allowed to hydrate in HEPES buffer saline (10 mM HEPES, 150 mM NaCl, pH 7.4). This suspension was extruded over polycarbonate membranes with 0.1 μm pore size using the Avanti Mini-Extruder. The liposomes (5 mg/mL) were incubated with MakA (500 nM), MakB (500 nM), MakE (500 nM) or the MakA/B/E tripartite complex (1:1:1) at 37 °C for 2 h. At the end of the experiment, the reaction mixture was crosslinked with Glutaraldehyde (0.05 %) for 10 min at 37 °C followed by the addition of stop solution Tris-HCl (200 mM, pH 6.8). Subsequently, the reaction mixture was centrifuged at 21,500 x *g* for 30 min. Liposome bound complexes were washed twice with the reaction buffer and loaded to the gel in the SDS-PAGE loading buffer. Liposome bound Mak proteins were detected using Mak proteins specific antibodies.

Transmission electron microscopy

For liposomes, negative staining samples were added to glow discharged 300 mesh copper grids with a thin film of carbon (Ted Pella, Redding, CA) washed twice with MQ water and negatively stained with 1.5% uranyl acetate solution (EMS (Hatfield, PA). Grids were examined with Talos L120C (FEI, Eindhoven, The Netherlands) operating at 120kV. Micrographs were acquired with a Ceta 16M CCD camera (FEI, Eindhoven, The Netherlands) using TEM Image & Analysis software ver. 4.17 (FEI, Eindhoven, The Netherlands).

Analysis of the *mak* gene clusters in different *Vibrio* genomes

Sequences of the *mak* operons were retrieved from GenBank (E-value =0), based on searching with the *mak* gene cluster of *V. cholerae* O1 biovar El Tor str. N16961 chromosome 2 (accession number NC_002506) together with their flanking ORFs against the fully sequenced bacterial genomes using BLASTN. Sequences were aligned using the MUSCLE alignment algorithm, and the phylogenetic relationships among different *mak* gene clusters in the retrieved dataset were estimated based on the Maximum Likelihood (ML) method implemented in MEGA-X (25). The ML model of gamma distribution with invariant sites (G+I)

was applied. The test of the clades was evaluated by 500 bootstrap replicates of Nearest-Neighbor-Interchange ML heuristic method.

Supplementary information

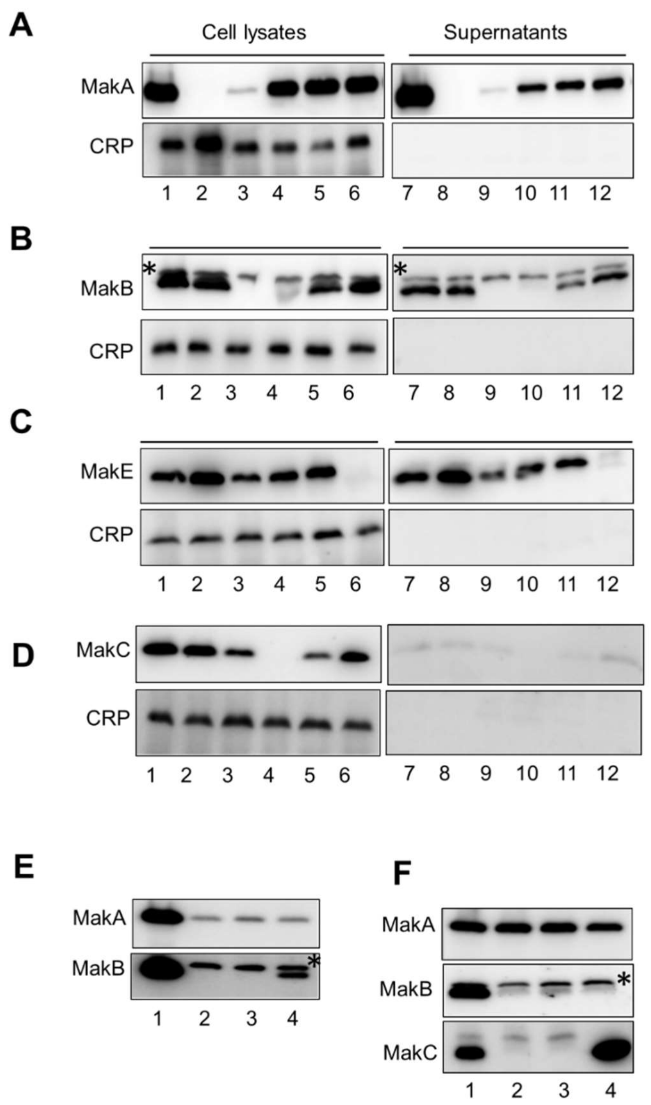

Supplementary Fig. 1: *Vibrio cholerae* secretion of Mak proteins.

(A-D) Western immunoblot detection of Mak protein expression and secretion from *V. cholerae* O1 El Tor strain A1552. Samples of bacterial cell lysates (lanes 1-6) and culture supernatants (lanes 7-12) were subjected to immunodetection with antisera raised against MakA (A), MakB (B), MakE (C), and MakC (D) respectively. Lanes 1 and 7, wild type; lanes 2 and 8, Δ*makA*; lanes 3 and 9, Δ*makB*; lanes 4 and 10, Δ*makC*; lanes 5 and 11, Δ*makD;* lanes 6 and 12, Δ*makE.* An additional band, marked with an asterisk, representing an unknown protein appeared in the case of the anti-MakB immunoblots. The cytoplasmic protein CRP was used as a reference and non-secreted protein control. Data are representative of three independent experiments. ***(E)*** Western blot analysis of whole cell lysates for MakA and MakB expression. Lane 1, wild-type, lane 2, Δ*makB*; lane 3, Δ*makB*/pvector, lane 4, Δ*makB*/p*makB.* The asterisk indicates a nonspecific band detected by the anti-MakB antiserum. ***(F)*** Western blot analysis of MakA, MakB and MakC expression. Lane 1, wild-type; lane 2, Δ*makC*; lane 3, Δ*makC/*pvector; lane 4, Δ*makC/*pmakC. The asterisk indicates a nonspecific band detected by the anti-MakB antiserum.

Supplementary information

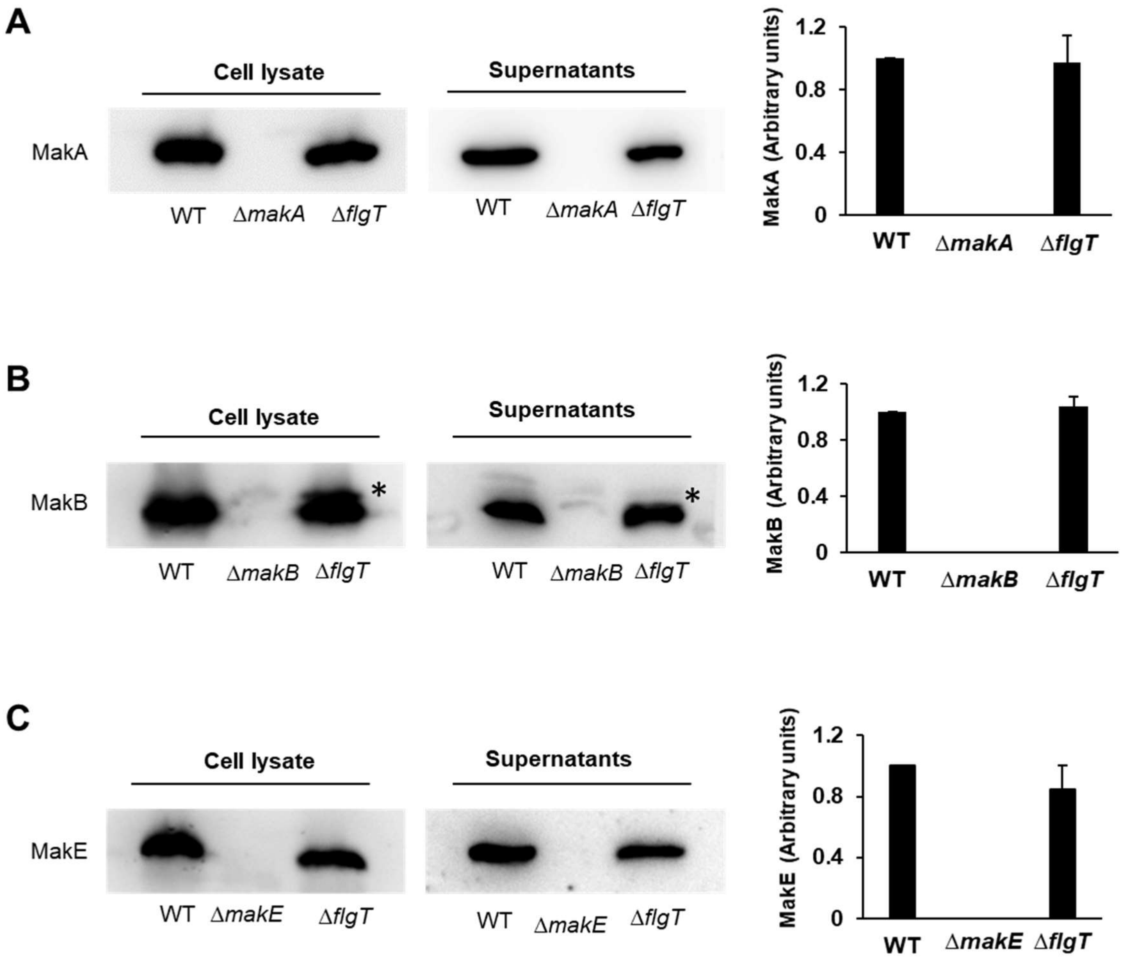

Supplementary Fig. 2: *Vibrio cholerae* expression and secretion of the MakA/B/E proteins are not affected by lack of FlgT.

Western blot analysis of Mak protein expression and secretion from *V. cholerae* O1 El Tor strain A1552 and Δ*flgT*. Samples of bacterial cell lysates or culture supernatants were subjected to immunodetection with antisera raised against MakA (**A**), MakB (**B**) and MakE (**C**), respectively. Samples from the respective Δ*mak* gene mutant derivatives of *V. cholerae* O1 El Tor strain are shown in the middle lane. Histograms to the right represent quantification of the Mak proteins. In (**B**), the asterisk indicates an unidentified protein also detected by the MakB antiserum. Data are representative of two independent experiments.

Supplementary information

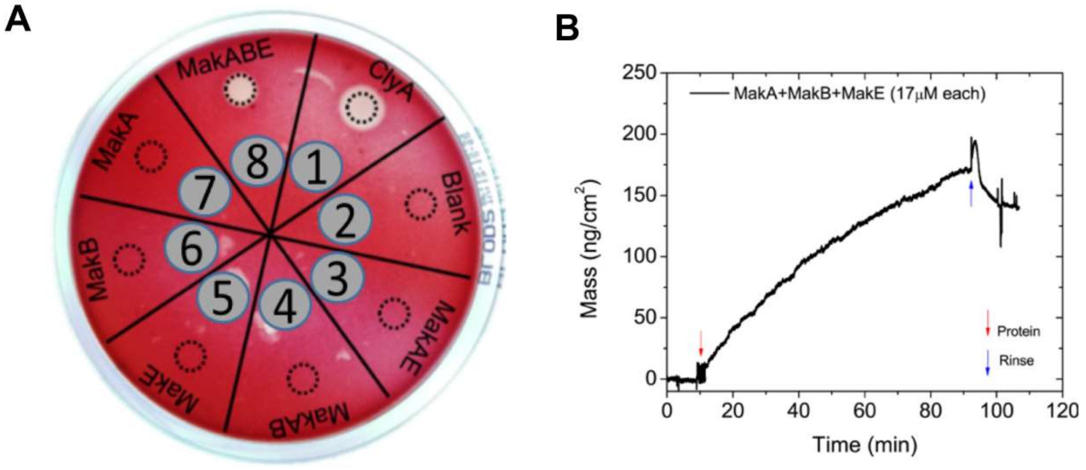

Supplementary Fig. 3: The tripartite MakA/B/E cytotoxin binds to red blood cell and induces hemolysis

(A) Test of hemolysis activity of Mak proteins on blood agar. Samples (5 µl) of protein solutions were spotted onto an agar plate containing horse erythrocytes. The plate was incubated overnight at 37 °C, and a zone of clearance indicated lysis of the erythrocytes. The spots (marked by a dotted ring) were: 1) The cytolytic *E. coli* protein ClyA (100 nM) used as positive control; 2) No protein; 3) MakA+MakE (250 nM each); 4) MakA+MakB (250 nM each); 5) MakE (250 nM); 6) MakB (250 nM); 7) MakA (250 nM); 8) MakA+MakB+MakE (250 nM each).

(B) Quartz Crystal Microbalance with dissipation (QCM-D) data confirming the binding of a mixture of MakA, MakB and MakE (17 µM each) to a supported lipid bilayer made from the synthetic lipid mixture (SLM). The frequency shift of the third overtone was converted into a mass value using the Sauerbrey equation. The addition of the protein is marked with a red downwards arrow and onset of rinsing with a buffer with a blue upwards arrow.

Supplementary information

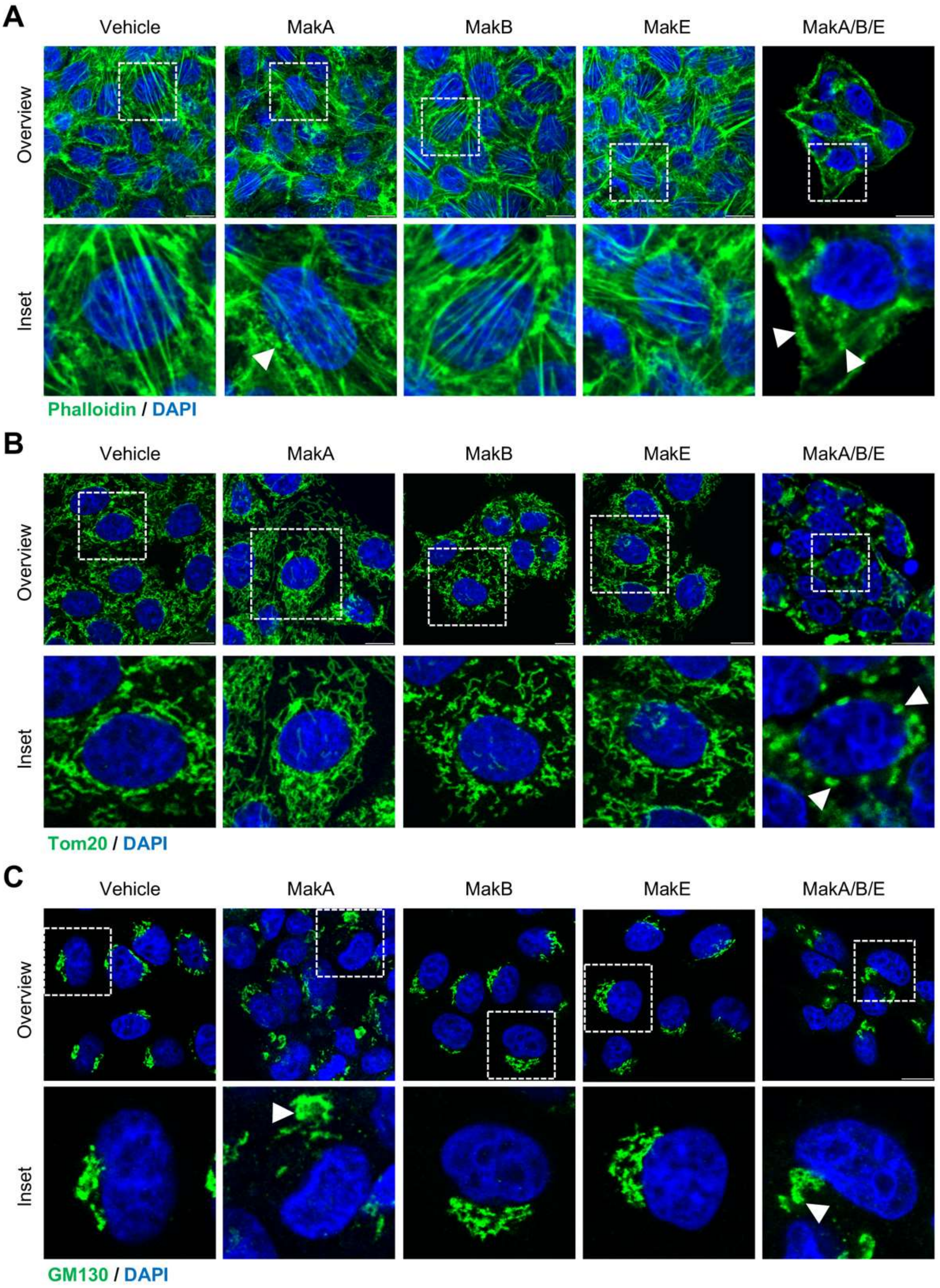

Supplementary information

D

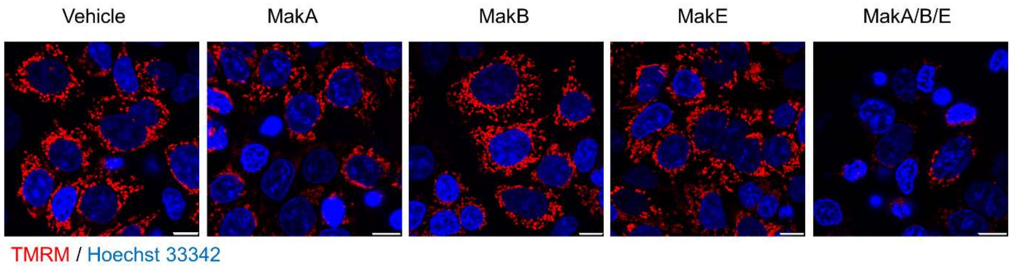

E

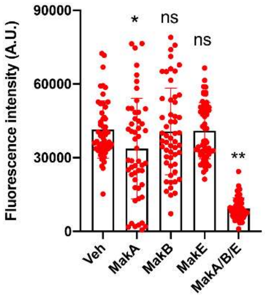

Supplementary Fig. 4: Effect of the tripartite MakA/B/E cytotoxin or its individual component, MakA, MakB, or MakE, on intracellular structures and organelles.

Caco-2 or HCT8 cells treated with an equal volume of vehicle, MakA (250 nM), MakB (250 nM), MakE (250 nM) or MakA/B/E cytotoxin (250 nM, equimolar concentration) for 24 h were examined by confocal laser scanning microscopy. Nuclei were counterstained with DAPI or Hoechst 33342. Scale bars represent 10 µm.

(A) Effect on actin filaments. Caco-2 cells were stained with Phalloidin Fluorescein isothiocyanate (FITC) to visualize actin filaments. Arrowheads (white) indicate disruption of actin filaments. ***(B)*** Effect on the Golgi apparatus. Caco-2 cells were stained using a cis-Golgi marker, GM130. Arrowhead (white) indicates changes in the cellular distribution of the Golgi complexes. ***(C)*** Effect on mitochondria in Caco-2 cells. Immunofluorescence detection was performed with antibodies against Tom20 to visualize mitochondria. Arrowhead (white) indicate swelling of mitochondria. ***(D)*** HCT8 cells treated with vehicle, individual components of the tripartite complex or MakA/B/E (24 h) and stained with mitochondrial potential marker, TMRM (250 nM, 30 min). Scale bars represent 10 µm. ***(E)*** Quantification of fluorescence intensity displayed upon treatment of HCT8 cells as shown in (D). Bar graphs show mean ± s.e.m. Data points represent fluorescence intensity of 51-57 individual cells.

Supplementary information

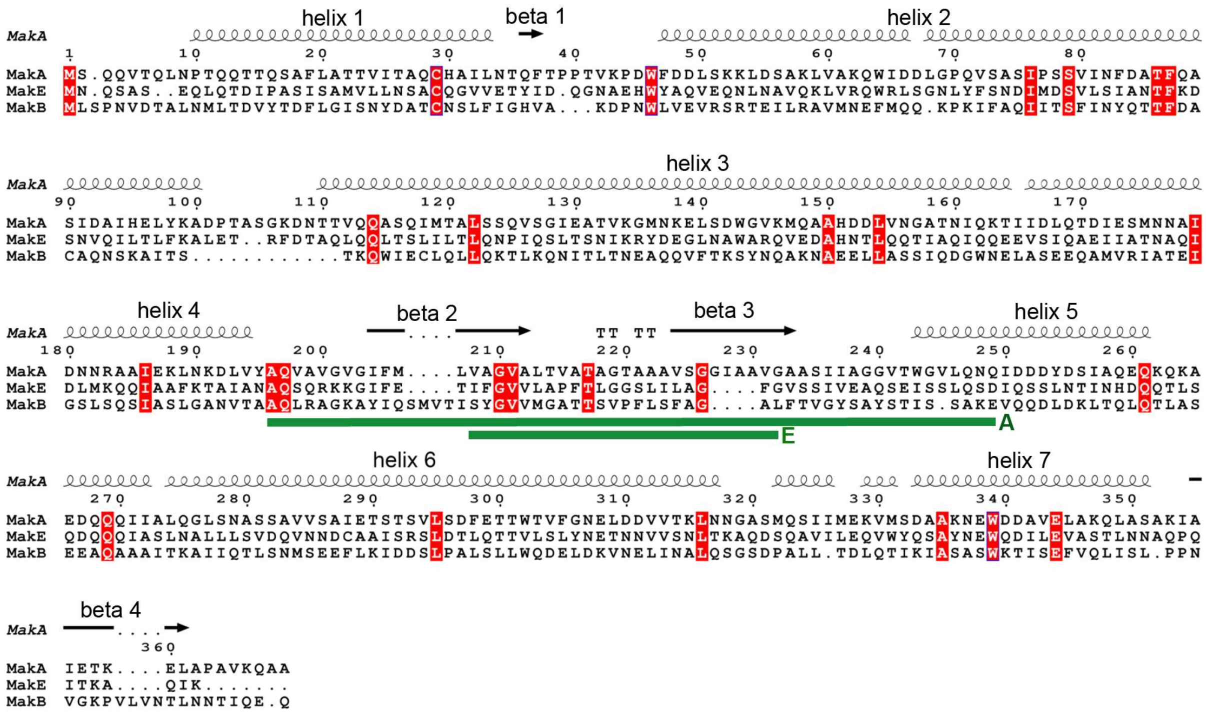

Supplementary Fig. 5: Amino acids sequence comparison between MakA, MakE and MakB.

Identical residues are highlighted in red. Secondary structure elements, based on the MakA structure, are indicated above the sequences. The residues in MakA and MakE predicted to be transmembrane helices are indicated with green bars below the sequence.

Supplementary information

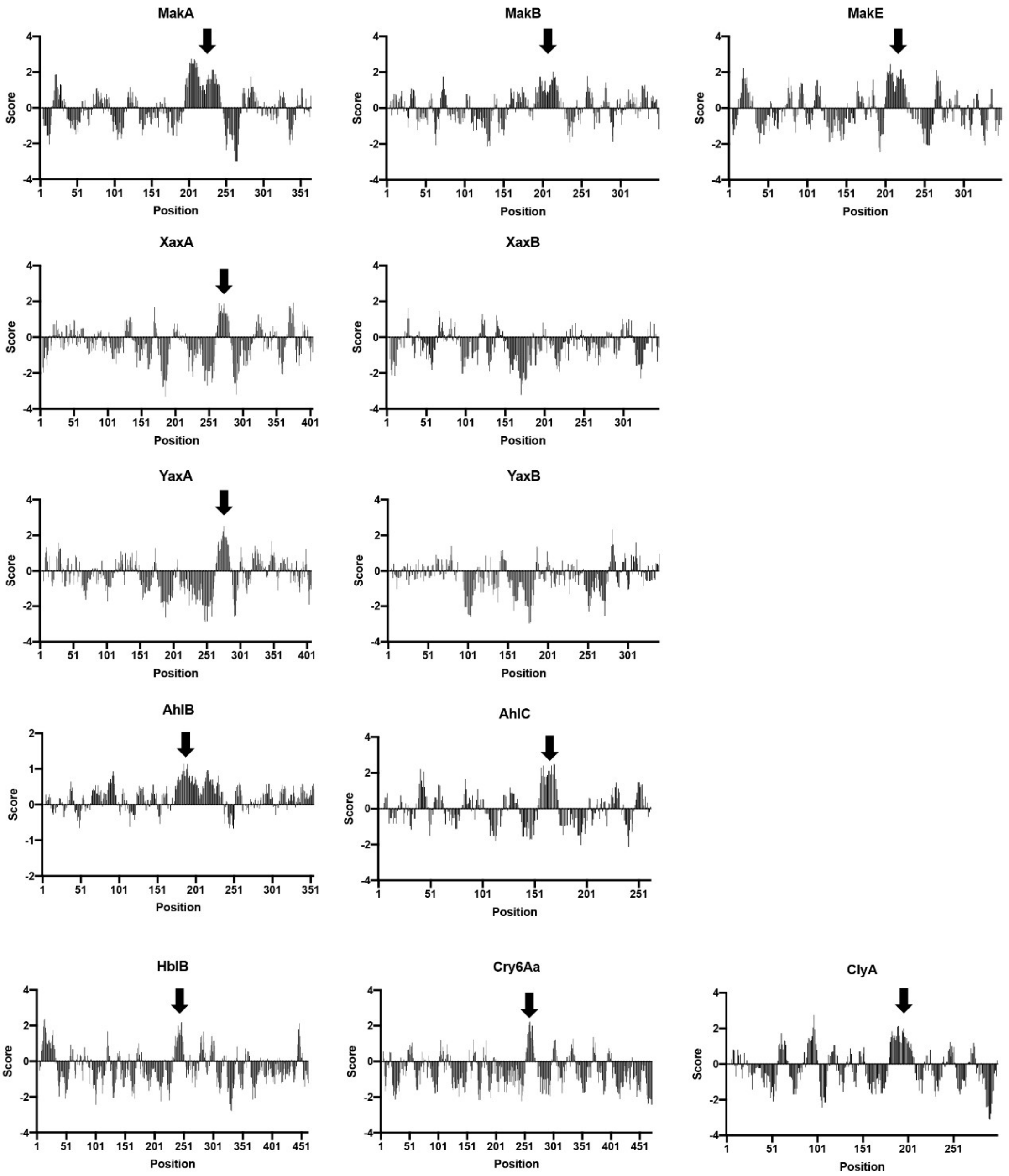

Supplementary Fig. 6: Kyte and Doolittle hydropathy plots.
Kyte and Doolittle plots were generated using ProtScale for structurally related proteins to MakA, MakB and MakE, identified by the Phyre2 database. The black arrow indicates predicted transmembrane regions.

Supplementary information

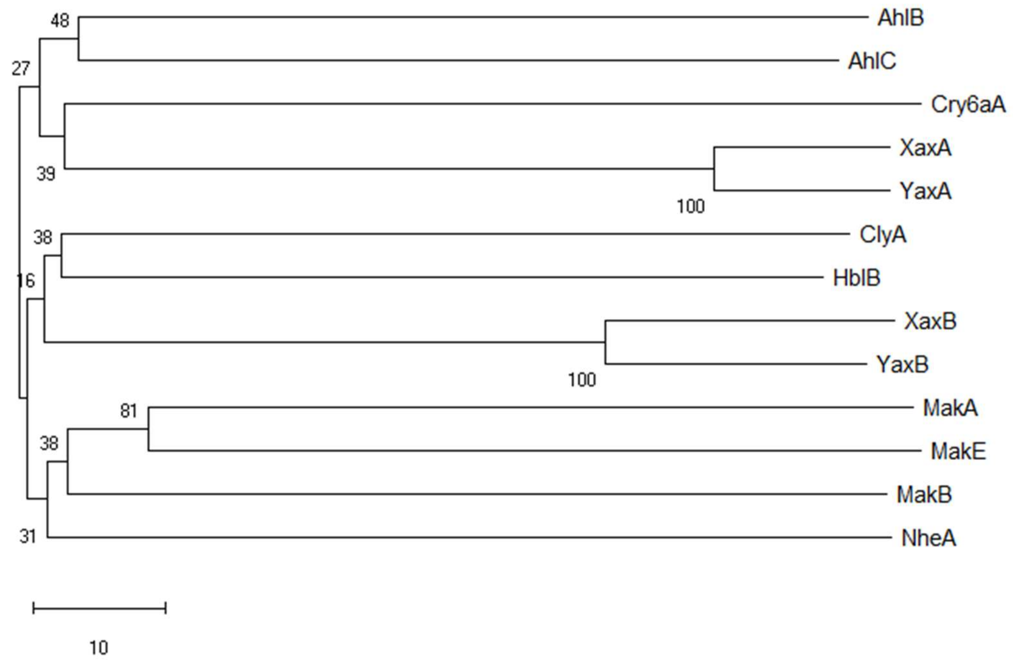

Supplementary Fig. 7. Evolutionary relationships of Mak and other bacterial toxins. Dendrogram showing hierarchical relationships between the structurally similar MakA, MakB, MakE and other ClyA family protein toxins using the Neighbor-Joining method (the bootstrap test was done with 500 replicates).

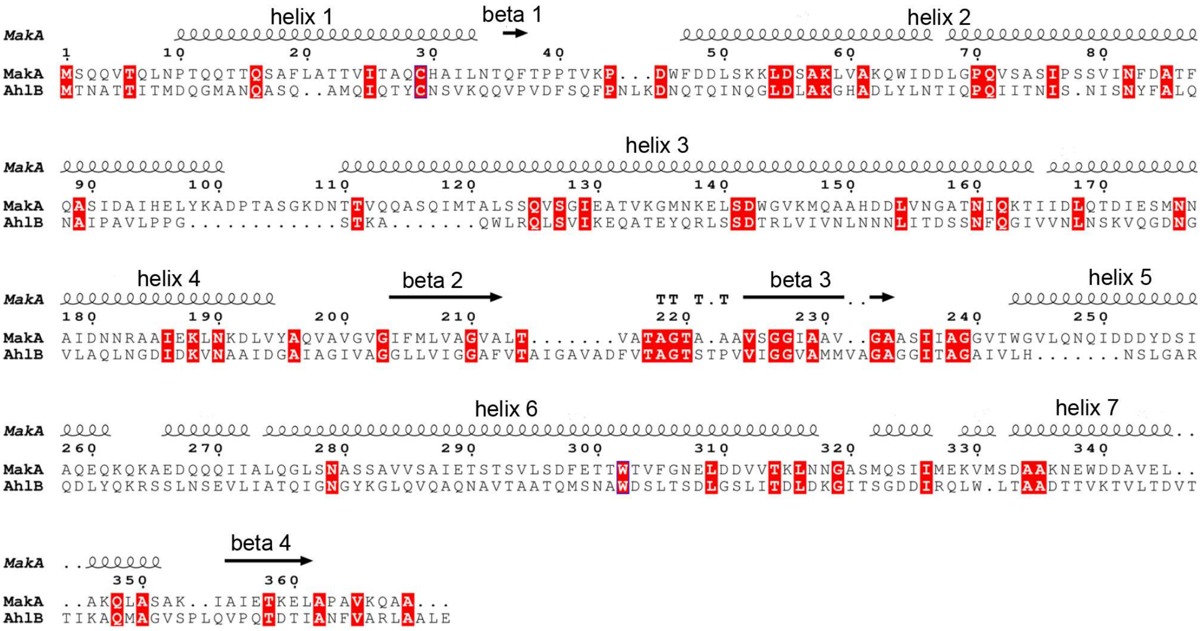

Supplementary Fig. 8: Amino acids sequence alignment of MakA and AhlB. Identical residues are highlighted in red. Secondary structure elements, based on the MakA structure, are indicated above the sequences.

Supplementary information

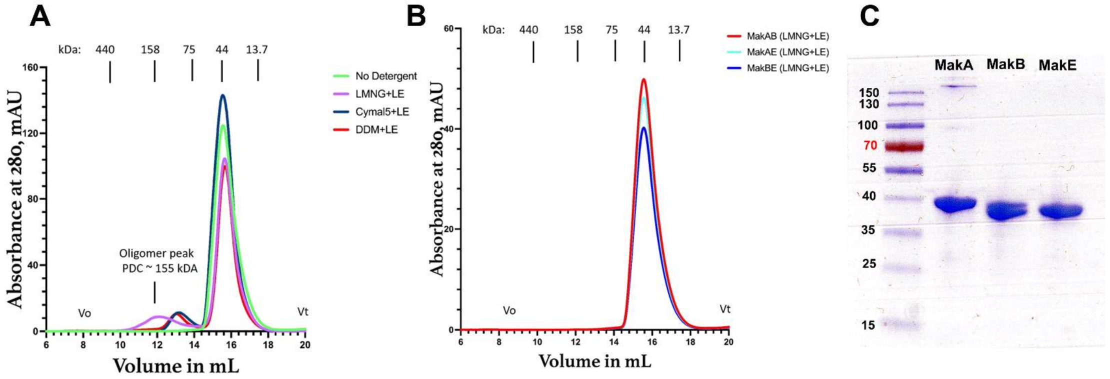

Supplementary Fig. 9: **Analysis of MakA/B/E proteins.**
(A) Gel filtration analysis of an equimolar (250 nM) mixture of MakA/B/E proteins incubated in absence or in the presence of selected detergents. *Gren* (no detergent), *red* (DDM+LE), *blue* (cymal5+LE), *purple* (LMNG+LE). Upon addition of DDM+LE and cymal5+LE, the monomeric MakA/B/E proteins immediately associated, leading to a shift in the elution volume from 15.6 mL to 13.0 mL. The LMNG+LE detergent-bound MakA/B/E monomers slowly converted to higher order oligomers eluting at 12.1 mL. As shown in the corresponding gel filtration trace (*purple*), the LMNG+LE resulted in a predominant oligomer peak at approximately 155 kDa. V_0 indicates void volume- V_t indicates the total liquid volume of the gel filtration column. ***(B)*** Gel filtration analysis of bipartite combinations (250 mM protein) performed in buffer +LMNG+LE. ***(C)*** SDS-PAGE and Coomassie Blue staining analysis of the recombinant MakA/B/E proteins used in this study.

Supplementary information

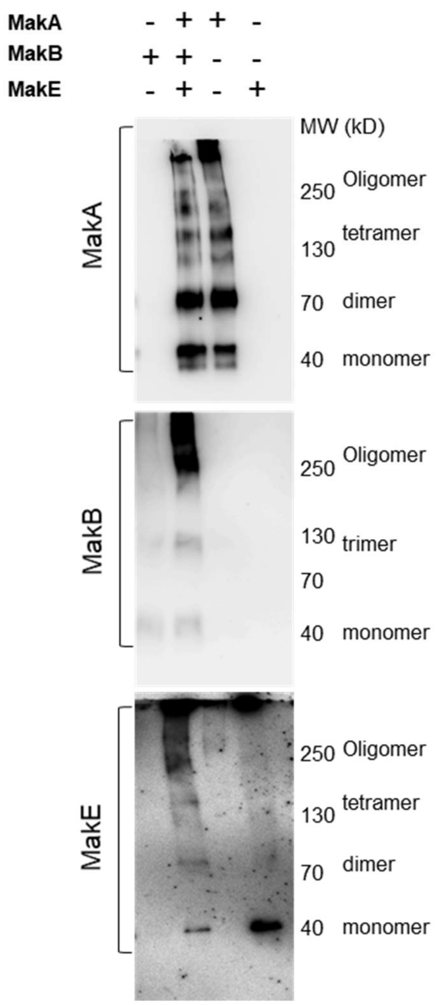

Supplementary Fig. 10: The MakA/B/E tripartite oligomerizes after binding with liposomes

Liposomes prepared from *E. coli* total lipid extract were incubated with MakA/BE tripartite (1:1:1, 500 nM each protein) or its individual components, MakA, MakB, or MakE (500 nM). The pull-down assay including a crosslinking step was performed as described in Materials and Methods. The liposome bound proteins were detected with Mak protein-specific antiserum. All proteins are detected in oligomeric form when all proteins were incubated with liposomes together. MakA binds to the liposomes on its own and polymerizes. MakB does not interact with liposomes in the absence of the other Mak proteins. MakE interacts with the membrane by itself but does not form oligomers.

Supplementary information

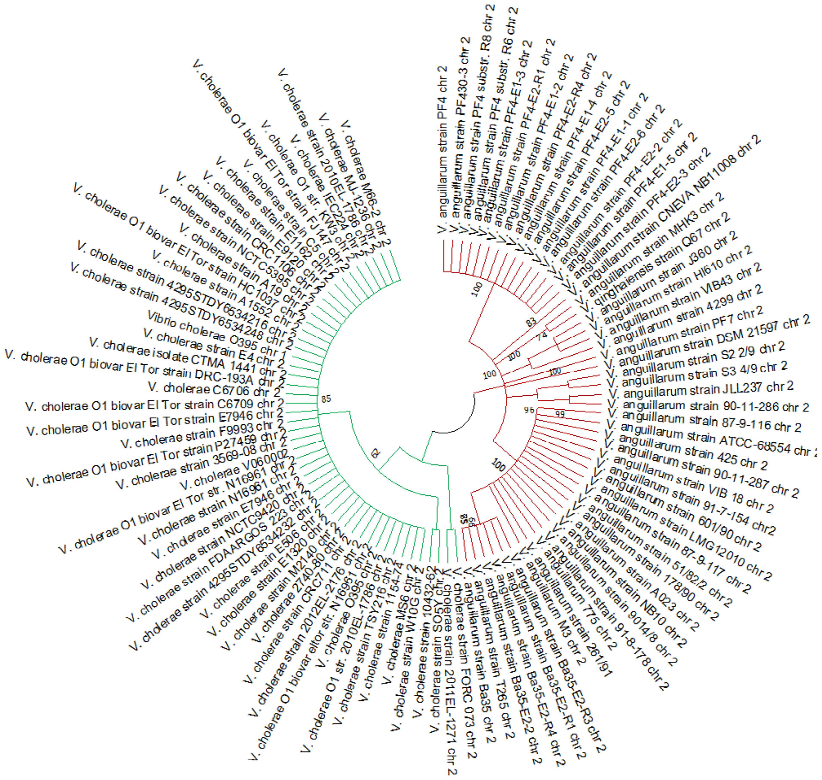

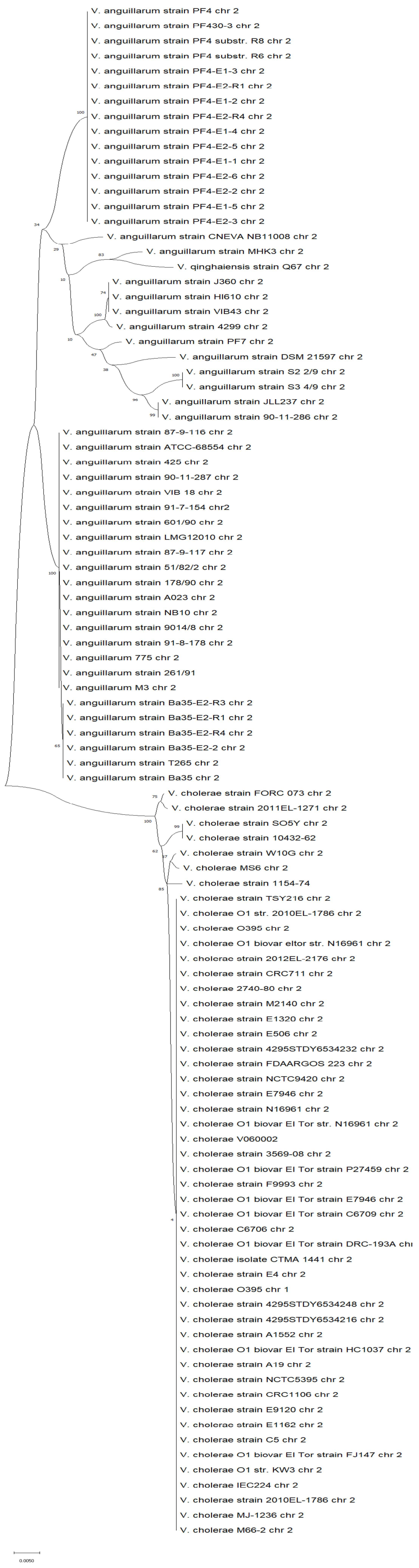

Supplementary information

Supplementary Fig. 11: Phylogenetic tree based on the *mak* gene cluster sequences located on the genomes of the *Vibrio* species shown in **Supplementary information Table S2**. **(A)** Circular tree which depicts the two main groups of the *mak* gene clusters: the first one of *V. cholerae* (in green) and the second one of the *V. anguillarum* group (in red). **(B)** Traditional phylogenetic tree indicating the degree of divergence with evolutionary distance indicated. The trees were constructed using the Maximum Likelihood method. The number of each node represents the bootstrap value derived from 500 replicates.

Supplementary information

Supplementary Table 1. Data collection and refinement statistics

	MakE SeMet	MakE native	MakB SeMet	MakB native
**Data collection**				
Space group	$P2_12_12_1$	$P2_1$	$P2_12_12_1$	$P2_12_12_1$
Cell dimensions				
a, *b*, *c* (Å)	66.6, 94.3, 116.6	56.9, 94.9, 63.6	53,2 54.2, 120.7	53,3 54.2, 120.8
α, β, γ (°)	90, 90, 90	90, 110.4, 90	90, 90, 90	90, 90, 90
Resolution (Å)*	49.3-2.60 (2.71-2.60)	49.2-1.98 (2.05-1.98)	49.5-1.92 (1.99-1.92)	48.8-2.10 (2.16-2.10)
R_{merge}*	0.141 (1.652)	0.0.086 (0.998)	0.075 (1.951)	0.097 (0.860)
$I / \sigma I$*	17.9 (2.0)	9.6 (1.2)	15.7 (1.4)	9.0 (1.9)
Completeness (%)	99.6(90.5)	99.9(100)	100(100)	99.8(99.5)
Redundancy	25.2(25.4)	4.5(4.4)	13.0(13.3)	6.5(6.5)
CC(1/2)*	1.00(0.836)	1(0.572)	0.999(0,778)	0.995(0.816)
Molecules in a.u.[a]	2	2	1	1
**Refinement**				
Resolution (Å)		49.2-1.98		60.41-2.10
No. reflections (work/test)		44086(2000)		20016(1056)
R_{work} / R_{free}		0.177(0.235)		0.215(0.261)
No. atoms				
Protein		5371		5474

Supplementary information

Ligand/ion		14 ($4Ni^{2+}$, $2SO_4^{2-}$)		10 (2 SO_4^{2-})
Water		412		61
B-factors (Å^2)				
Protein		41.3		48.4
Ligand/ion		79.4		61.04
Water		43.1		48.36
R.m.s. deviations				
Bond lengths (Å)		0.013		0.003
Bond angles (°)		1.14		0.55
PDB code		6TAO		6T8D

For each structure, diffraction data were collected on a single crystal.

*Values in parentheses are for the highest-resolution shell.

[a]Abbreviation: a.u. asymmetric unit.

Supplementary information

Supplementary Table 2. *Vibrio* strains with completely sequenced genomes that have the *mak* gene cluster

[1]*Vibrio* Strain<br>O serogroup & biovar if known	[2]Chromosome	Coordinates in the genome	[3]Orientation of *mak* genes	[4]Accession number
V. cholerae O1 biovar El Tor strain A1552	Chr. 2	832257-837904	→	CP028895.1
V. cholerae O1 biovar El Tor strain C6709	Chr. 2	832241-837888	→	CP047298.1
V. cholerae O1 biovar El Tor strain P27459	Chr. 2	835429-841076	→	CP047300.1
V. cholerae O1 biovar El Tor strain DRC-193A	Chr. 2	807261-812908	→	CP047302.1
V. cholerae O1 biovar El Tor strain E7946	Chr. 2	832887-838534	→	CP047304.1
V. cholerae O1 biovar El Tor strain E7946	Chr. 2	830081-835728	→	CP024163.1
V. cholerae O1 biovar El Tor strain CTMA_1441	Chr. 2	821464-827198	→	CP047060.1
V. cholerae strain F9993	Chr. 2	956732-962379	←	CP046841.1
V. cholerae O1 biovar El Tor strain C6706	Chr. 2	667286-672933	←	CP046845.1
V. cholerae O1 biovar El Tor strain 3569-08	Chr. 2	174549-180196	←	CP046743.1
V. cholerae O1 biovar El Tor strain N16961	Chr. 2	831898-837545	→	LT906615.1
V. cholerae O1 biovar El Tor strain N16961	Chr. 2	831895-837542	→	AE003853.1
V. cholerae O1 biovar El Tor strain N16961	Chr. 2	834209-839857	→	CP028828.1
V. cholerae O1 biovar El Tor strain E4	Chr. 2	771425-777072	←	CP033513.1

V. cholerae O1 biovar El Tor strain V060002	Chr.	2149839-2155486	←	AP018677.1
V. cholerae O139 biovar El Tor strain 4295STDY6534248	Chr. 2	703431-709078	→	LT992493.1
V. cholerae O139 biovar El Tor strain 4295STDY6534232	Chr. 2	1013978-1019625	←	LT992489.1
V. cholerae O139 biovar El Tor strain 4295STDY6534216	Chr. 2	481479-487126	←	LT992487.1
V. cholerae O1 biovar El Tor strain FDAARGOS_223	Chr. 2	347303-352950	←	CP020407.2
V. cholerae O1 biovar El Tor strain HC1037	Chr. 2	532359-538006	→	CP026648.1
V. cholerae O1 biovar El Tor strain A19	Chr. 2	831898-837545	→	LT907990.1
V. cholerae O1 biovar El Tor strain NCTC9420	Chr. 2	827597-833244	→	CP013320.1
V. cholerae O1 biovar El Tor strain NCTC5395	Chr. 2	892952-898599	→	CP013318.1
V. cholerae O1 biovar El Tor strain M2140	Chr. 2	618070-623717	→	CP013316.1
V. cholerae O1 biovar El Tor strain E9120	Chr. 2	864606-870253	→	CP013314.1
V. cholerae O1 biovar El Tor strain E1320	Chr. 2	108643-114290	→	CP013312.1
V. cholerae O1 biovar El Tor strain E1162	Chr. 2	992794-998441	→	CP013310.1
V. cholerae O1 biovar El Tor strain E506	Chr. 2	852260-857907	→	CP013308.1
V. cholerae O1 biovar El Tor strain CRC1106	Chr. 2	859687-865334	→	CP013306.1
V. cholerae O1 biovar El Tor strain CRC711	Chr. 2	823844-829491	→	CP013304.1

Supplementary information

V. cholerae O1 biovar El Tor strain C5	Chr. 2	865423-871070	→	CP013302.1
V. cholerae O1 biovar El Tor strain 2740-80	Chr. 2	861068-866715	→	CP016325.1
V. cholerae O1 strain KW3	Chr. 2	807251-812898	→	CP006948.1
V. cholerae O1 biovar El Tor strain TSY216	Chr. 2	809852-815499	→	CP007654.1
V. cholerae O1 biovar El Tor strain FJ147	Chr. 2	806032-811679	→	CP009041.1
V. cholerae O1 biovar El Tor strain 2012EL-2176	Chr. 2	533667-539314	→	CP007635.1
V. cholerae O1 strain IEC224	Chr. 2	831720-837367	→	CP003331.1
V. cholerae O1 biovar El Tor strain 2010EL-1786	Chr. 2	532427-538074	→	CP003070.1
V. cholerae O1 biovar El Tor strain MJ-1236	Chr. 2	508290-513937	←	CP001486.1
V. cholerae O1 classical strain O395	Chr. 2	870719-876366	→	CP001236.1
V. cholerae O1 classical strain O395	Chr. 2	387576-393223	←	CP000626.1
V. cholerae O1 classical strain O395	Chr. 1	387576-393310	←	CP045718.1
V. cholerae O1 biovar El Tor strain M66-2	Chr. 2	805881-811528	→	CP001234.1
V. cholerae non-O1/O139 environmental isolate strain W10G	Chr. 2	788603-794251	→	CP053795.1
V. cholerae O1 biovar El Tor strain 2010EL-1786	Chr. 2	664915-670552	→	CP038179.2
V. cholerae O1 biovar El Tor strain MS6	Chr. 2	847066-852712	→	AP014525.1

Supplementary information

V. cholerae non-O1/O139 environmental isolate strain SO5Y	Chr. 2	814545-820160	→	CP053801.1
V. cholerae O27 strain 10432-62	Chr.	338019-343642	←	CP010812.1
V. cholerae strain FORC_073	Chr. 2	759334-765000	→	CP024083.1
V. cholerae O1 strain 2011EL-1271	Chr. 2	386300-391301	←	CP046838.1
V. cholerae O49 strain 1154-74	Chr.	2336425-2341366	←	CP010811.1
V. anguillarum O1 strain 425	Chr. 2	790251-794855	←	CP020533.1
V. anguillarum O1 strain 87-9-116	Chr. 2	7345-11949	←	CP021981.1
V. anguillarum O1 strain VIB 18	Chr. 2	543776-548380	←	CP011437.1
V. anguillarum O1 strain 90-11-287	Chr. 2	540749-545353	←	CP011476.1
V. anguillarum O1 strain 91-7-154	Chr. 2	540911-545515	←	CP010083.1
V. anguillarum O1 strain 601/90	Chr. 2	540787-545391	←	CP010077.1
V. anguillarum O1 strain 178/90	Chr. 2	540837-545441	←	CP011471.1
V. anguillarum O1 strain LMG12010	Chr. 2	536719-541323	←	CP011469.1
V. anguillarum O1 strain 87-9-117	Chr. 2	538384-542988	←	CP010047.1
V. anguillarum O1 strain 51/82/2	Chr. 2	539273-543877	←	CP010043.1
V. anguillarum O1 strain 9014/8	Chr. 2	539619-544223	←	CP010039.1
V. anguillarum O1 strain A023	Chr. 2	541898-546502	←	CP010037.1

Supplementary information

V. anguillarum O1 strain 91-8-178	Chr. 2	543326-547930	←	CP010035.1
V. anguillarum O1 strain NB10	Chr. 2	7344-11948	←	LK021129.1
V. anguillarum O1 strain M3	Chr. 2	543797-548401	←	CP006700.1
V. anguillarum O1 strain 775	Chr. 2	543821-548425	←	CP002285.1
V. anguillarum O1 strain Ba35-E2-R3	Chr. 2	542397-547001	←	CP031528.1
V. anguillarum O1 strain Ba35-E2-R1	Chr. 2	544119-548723	←	CP031524.1
V. anguillarum O1 strain Ba35-E2-R4	Chr. 2	542233-546837	←	CP031532.1
V. anguillarum O1 strain Ba35-E2-2	Chr. 2	543973-548577	←	CP031520.1
V. anguillarum O1 strain T265	Chr. 2	543438-548042	←	CP010041.1
V. anguillarum O1 strain Ba35	Chr. 2	543336-547940	←	CP010031.1
V. anguillarum O3 strain PF4-E2-3	Chr. 2	859280-863881	→	CP031493.1
V. anguillarum O3 strain PF4-E1-5	Chr. 2	876563-881164	→	CP031486.1
V. anguillarum O3 strain PF4-E2-2	Chr. 2	859282-863883	→	CP031491.1
V. anguillarum O3 strain PF4-E2-5	Chr. 2	859284-863885	→	CP031497.1
V. anguillarum O3 strain PF4-E2-6	Chr. 2	859284-863885	→	CP031499.1
V. anguillarum O3 strain PF4-E1-1	Chr. 2	874235-878836	→	CP031478.1
V. anguillarum O3 strain PF4-E1-4	Chr. 2	874234-878835	→	CP031484.1

Supplementary information

V. anguillarum O3 strain PF4-E2-R4	Chr. 2	859284-863885	→	CP031495.1
V. anguillarum O3 strain PF4-E1-2	Chr. 2	874235-878836	→	CP031480.1
V. anguillarum O3 strain PF4-E2-R1	Chr. 2	859287-863888	→	CP031489.1
V. anguillarum O3 strain PF4-E1-3	Chr. 2	874234-878835	→	CP031482.1
V. anguillarum O3 strain PF4 substr. R6	Chr. 2	860025-864626	→	CP023432.1
V. anguillarum O3 strain PF4 substr. R8	Chr. 2	860021-864622	→	CP023292.1
V. anguillarum O3 strain PF4	Chr. 2	860022-864623	→	CP023290.1
V. anguillarum O3 strain PF4 substr. R4	Chr. 2	860025-864626	→	CP023288.1
V. anguillarum O3 strain PF430-3	Chr. 2	577775-582379	←	CP011467.1
V. anguillarum O3 strain PF7	Chr. 2	343423-348025	→	CP011465.1
V. anguillarum O2 strain J360	Chr. 2	175977-180582	←	CP034673.1
V. anguillarum O1 strain VIB43	Chr. 2	7356-11961	←	CP023055.1
V. anguillarum O2a strain HI610	Chr. 2	582185-586790	←	CP011463.1
V. anguillarum non-O1/O2/O3/O4/O5 strain MHK3	Chr. 2	324177-328777	→	CP022469.1
V. anguillarum O3 strain CNEVA NB11008	Chr. 2	7355-11962	←	CP022104.1
V. anguillarum O2b strain 4299	Chr. 2	542862-547467	←	CP011459.1
V. anguillarum O1 strain S2 2/9	Chr. 2	529358-533980	←	CP011473.1

V. anguillarum O1 strain S3 4/9	Chr. 2	7355-11977	←	CP022100.1
V. anguillarum O2 strain DSM 21597	Chr. 2	576229-580860	←	CP010085.1
V. anguillarum O1 strain JLL237	Chr. 2	7354-11220	←	CP022102.1
V. anguillarum O1 strain 90-11-286	Chr. 2	714386-718252	←	CP011461.1
V. anguillarum O1 strain 261/91	Chr. 2	544444-547945	←	CP010033.1
V. anguillarum O1 strain ATCC-68554	Chr. 2	983079-989192	→	CP023209.1
V. qinghaiensis strain Q67	Chr. 2	28543-33164	←	CP022742.1

1. Strains represented more than once in the table have undergone repeated sequencing of their genomes and were reported independently. Identification of the *mak* gene cluster was therefore indicated for each report using the corresponding accession number.
2. Abbreviations: Chr. 1 = chromosome 1; Chr. 2 = chromosome 2; Chr. = genome composed of only one chromosome.
3. → indicates that the gene cluster is oriented in the clockwise direction relative to the replication origin and ← indicates that the gene cluster is oriented in the counterclockwise direction.
4. GenBank: https://www.ncbi.nlm.nih.gov/genbank/

Supplementary information

Supplementary Table 3: Bacterial strains and plasmids used in this study

Strain/<br>plasmid	Description/relevant characteristics	Reference / Source
Vibrio cholerae		
Vibrio cholerae A1552: serogroup O1, biotype El Tor, Rif^r		Yildiz et al., 1998(26)
MDS002	A1552Δ*makD*	Dongre et al., 2018(1)
MDS003	A1552Δ*makC*	Dongre et al., 2018(1)
MDS004	A1552Δ*makB*	Dongre et al., 2018(1)
MDS005	A1552Δ*makA*	Dongre et al., 2018(1)
MDC008	A1552Δ*makE*	This study
MDS013	A1552Δ*flhA*	Dongre et al., 2018(1)
TAKA-23	A1552Δ*flgT*	This Study
MDC003	A1552Δ*makB*/p*makB*	Dongre et al., 2018(1)
MDC002	A1552Δ*makC*/p*makC*	Dongre et al., 2018(1)
Escherichia coli		
DH5α	*F$^-$ Φ80lacZΔM15 Δ(lacZYA-argF) U169 recA1 endA1 hsdR17(r_K^-, m_K^+) phoA supE44 thi-1 gyrA96 relA1 λ$^-$*	Grant et al., 1985(27)
BL21(DE3)	*fhuA*2 [*lon*] *ompT gal* (λ DE3) [dcm] Δ*hsdS*<br>λ DE3 = λ s*BamHI*o Δ*EcoRI-B* int::(*lacI*::PlacUV5::*T7 gene1*) i21Δ*nin5*	Studier and Moffatt,1986(28)
SM10λpir	*thi thr leu tonA lacY supE recA*::RP4-2 Tc::Mu Km λpir	Miller et al., 1987(29)
MDS611	Top10/pBAD18; Ap^r	Dongre et al., 2018(1)
AA1	Top10/p*makB$^+$A$^+$E$^+$*	This Study
AA2	Top10/p*makB*$^+$$A_{L193T,L246T}$$E^+$	This Study
AA3	Top10/p*makB*$^+$$A_{L193T,L207T,L213T,L246T}$$E^+$	This Study
**Plasmids**		
pCVD442	Suicide plasmid; R6K ori, mobRP4, bla, *sacB*; Ap^r	Donnenberg et al., 1991(3)

Supplementary information

pMDC008	pCVD442::Δ*makE*	This Study
pTAKA-23	pCVD442::Δ*flgT*	This Study
pMDC003	pBAD18-*makB*	Dongre et al., 2018(1)
pMDC002	pBAD18-*makC*	Dongre et al., 2018(1)
pAA1	pBAD18- $makB^{+}A^{+}E^{+}$	This Study
pAA2	pBAD18-$makB^{+}A_{L193T,L246T}E^{+}$	This Study
pAA3	pBAD18-$makB^{+}A_{L193T,L207T,L213T,L246T}E^{+}$	This Study

Supplementary information

Supplementary Table 4. Primers used in this study

Sequence 5'→3'	Primer	For construction of
atagagctccctcgttatttcactgtagag	makB-pBAD18-5Sac1	$makB^{+}A^{+}E^{+}$
gtctctagattacttgatttgagcttttgttat	makE-pBAD18-3Xba1	$makB^{+}A^{+}E^{+}$
gtagtcatcatcaatttgattttgcgttacgccccatgt aacgccaccag	makA_t736a_t737c_a738g_F	$makB^{+}A_{L193T,L246T}E^{+}$
ctggtggcgttacatggggcgtaacgcaaaatca aattgatgatgactac	makA_t736a_t737c_a738g_R	$makB^{+}A_{L193T,L246T}E^{+}$
caacgcctgcgacggtcataaagatgccaacac caac	makA_c619a_t620c_F	$makB^{+}A_{L193T,L246T}E^{+}$
gttggtgttggcatctttatgaccgtcgcaggcgttg	makA_c619a_t620c_R	$makB^{+}A_{L193T,L246T}E^{+}$
gtagtcatcatcaatttgattttgcgttacgccccatgt aacgccaccag	makA_t736a_t737c_a738g_F	$makB+A_{L193T,L207T,L213T,L246T}E+$
ctggtggcgttacatggggcgtaacgcaaaatca aattgatgatgactac	makA_t736a_t737c_a738g_R	$makB+A_{L193T,L207T,L213T,L246T}E+$
ccgcaacttgtgcatagaccgtatctttattcagtttct caatgg	makA_c577a_t578c_F	$makB+A_{L193T,L207T,L213T,L246T}E+$
ccattgagaaactgaataaagatacggtctatgca caagttgcgg	makA_c577a_t578c_R	$makB+A_{L193T,L207T,L213T,L246T}E+$
cggttgcgacggtagtcgcaacgcctgcga	makA_c637a_t638c_F	$makB+A_{L193T,L207T,L213T,L246T}E+$
tcgcaggcgttgcgactaccgtcgcaaccg	makA_c637a_t638c_R	$makB+A_{L193T,L207T,L213T,L246T}E+$
caacgcctgcgacggtcataaagatgccaacac caac	makA_c619a_t620c_F	$makB+A_{L193T,L207T,L213T,L246T}E+$
gttggtgttggcatctttatgaccgtcgcaggcgttg	makA_c619a_t620c_R	$makB+A_{L193T,L207T,L213T,L246T}E+$
cattctagaagctgcagtctccggcggtatc	makE-A F	Δ*makE*
cccatccactataaactaacaagaatagaaggg ggcgtaacc	makE-B R	Δ*makE*
tgttagtttatagtggatgggaagtaattgatttcgct ccag	makE-C F	Δ*makE*
cattctagactgatgggaaagctcattaaac	makE-D R	Δ*makE*
cgctctagatgctgcgtcacttggattgaa	flgT-A F	Δ*flgT*

Supplementary information

cccatccactataaactaacatgcatgagccgatg gaagttgcaatctatgcagcggctaaga	flgT-B R	Δ*flgT*
tgttagtttatagtggatgggattcaaatcggcgatg tgatg	flgT-C F	Δ*flgT*
cgctctagaacagatattccgattgacctgcaggtc aatcggaatatctgt	flgT-D R	Δ*flgT*
gtaacggtctcccatgttaagcccaaatgttgatac	MakB-F	Cloning of recombinant MakB
gttacctcgagttactgttcttgaatggtgttatttag	MakB-R	Cloning of recombinant MakE
gtaactcatgaatcaatcagccagcgag	MakE-F	Cloning of recombinant MakE
gtaacaagcttacttgatttgagcttttgttatttg	MakE-R	Cloning of recombinant MakE

Supplementary information

References

1. Dongre M*, et al.* (2018) Flagella-mediated secretion of a novel Vibrio cholerae cytotoxin affecting both vertebrate and invertebrate hosts. *Communications Biology* 1(1):59.
2. Heidelberg JF*, et al.* (2000) DNA sequence of both chromosomes of the cholera pathogen Vibrio cholerae. *Nature* 406(6795):477-483.
3. Donnenberg MS & Kaper JB (1991) Construction of an eae deletion mutant of enteropathogenic Escherichia coli by using a positive-selection suicide vector. *Infect Immun* 59(12):4310-4317.
4. Doublie S (1997) Preparation of selenomethionyl proteins for phase determination. *Methods Enzymol* 276:523-530.
5. Kabsch W (2010) Xds. *Acta Crystallogr D Biol Crystallogr* 66(Pt 2):125-132.
6. Evans PR & Murshudov GN (2013) How good are my data and what is the resolution? *Acta Crystallogr D Biol Crystallogr* 69(Pt 7):1204-1214.
7. Winn MD*, et al.* (2011) Overview of the CCP4 suite and current developments. *Acta Crystallogr D Biol Crystallogr* 67(Pt 4):235-242.
8. Panjikar S, Parthasarathy V, Lamzin VS, Weiss MS, & Tucker PA (2005) Auto-rickshaw: an automated crystal structure determination platform as an efficient tool for the validation of an X-ray diffraction experiment. *Acta Crystallogr D Biol Crystallogr* 61(Pt 4):449-457.
9. Langer G, Cohen SX, Lamzin VS, & Perrakis A (2008) Automated macromolecular model building for X-ray crystallography using ARP/wARP version 7. *Nat Protoc* 3(7):1171-1179.
10. Afonine PV*, et al.* (2012) Towards automated crystallographic structure refinement with phenix.refine. *Acta Crystallogr D Biol Crystallogr* 68(Pt 4):352-367.
11. Skubak P & Pannu NS (2013) Automatic protein structure solution from weak X-ray data. *Nature communications* 4:2777.
12. Emsley P, Lohkamp B, Scott WG, & Cowtan K (2010) Features and development of Coot. *Acta Crystallogr D Biol Crystallogr* 66(Pt 4):486-501.
13. Vagin A & Teplyakov A (2010) Molecular replacement with MOLREP. *Acta Crystallographica Section D-Biological Crystallography* 66(Pt 1):22-25.
14. McCoy AJ (2007) Solving structures of protein complexes by molecular replacement with Phaser. *Acta Crystallographica Section D-Biological Crystallography* 63(Pt 1):32-41.
15. Winn MD, Isupov MN, & Murshudov GN (2001) Use of TLS parameters to model anisotropic displacements in macromolecular refinement. *Acta Crystallographica Section D-Biological Crystallography* 57(Pt 1):122-133.
16. McNicholas S, Potterton E, Wilson KS, & Noble ME (2011) Presenting your structures: the CCP4mg molecular-graphics software. *Acta Crystallographica Section D-Biological Crystallography* 67(Pt 4):386-394.
17. Waterhouse A*, et al.* (2018) SWISS-MODEL: homology modelling of protein structures and complexes. *Nucleic acids research* 46(W1):W296-W303.
18. Wilson JS*, et al.* (2019) Identification and structural analysis of the tripartite α-pore forming toxin of Aeromonas hydrophila. *Nature Communications* 10(1):2900.
19. Towbin H, Staehelin T, & Gordon J (1979) Electrophoretic transfer of proteins from polyacrylamide gels to nitrocellulose sheets: procedure and some applications. *Proc Natl Acad Sci U S A* 76(9):4350-4354.
20. Oscarsson J*, et al.* (1999) Molecular analysis of the cytolytic protein ClyA (SheA) from Escherichia coli. *Mol Microbiol* 32(6):1226-1238.
21. Wai SN*, et al.* (2003) Characterization of dominantly negative mutant ClyA cytotoxin proteins in Escherichia coli. *J Bacteriol* 185(18):5491-5499.
22. Schindelin J*, et al.* (2012) Fiji: an open-source platform for biological-image analysis. *Nat Methods* 9(7):676-682.

23. Wesen E, Jeffries GDM, Matson Dzebo M, & Esbjorner EK (2017) Endocytic uptake of monomeric amyloid-beta peptides is clathrin- and dynamin-independent and results in selective accumulation of Abeta(1-42) compared to Abeta(1-40). *Sci Rep* 7(1):2021.
24. Lorizate M*, et al.* (2013) Comparative lipidomics analysis of HIV-1 particles and their producer cell membrane in different cell lines. *Cell Microbiol* 15(2):292-304.
25. Kumar S, Stecher G, Li M, Knyaz C, & Tamura K (2018) MEGA X: Molecular Evolutionary Genetics Analysis across Computing Platforms. *Mol Biol Evol* 35(6):1547-1549.
26. Yildiz FH & Schoolnik GK (1998) Role of rpoS in stress survival and virulence of Vibrio cholerae. *J Bacteriol* 180(4):773-784.
27. Grant SG, Jessee J, Bloom FR, & Hanahan D (1990) Differential plasmid rescue from transgenic mouse DNAs into Escherichia coli methylation-restriction mutants. *Proc Natl Acad Sci U S A* 87(12):4645-4649.
28. Studier FW & Moffatt BA (1986) Use of bacteriophage T7 RNA polymerase to direct selective high-level expression of cloned genes. *J Mol Biol* 189(1):113-130.
29. Miller VL, Taylor RK, & Mekalanos JJ (1987) Cholera toxin transcriptional activator toxR is a transmembrane DNA binding protein. *Cell* 48(2):271-279.
